# Realizing renewable resilience: Lessons from the Middle East for the global energy transition

**DOI:** 10.1016/j.isci.2024.110058

**Published:** 2024-05-21

**Authors:** Ansari Aadil Shahzad, Abdullahi Bamigbade, Krishiv Gandhi, Juan David Barbosa, Andrei Sleptchenko, Harry Nicholas Apostoleris, Sgouris Sgouridis

**Affiliations:** 1 Dubai Electricity & Water Authority Research & Development Center, Dubai, UAE; 2 Tandon School of Engineering, New York University, New York, NY, USA; 3 Khalifa University of Science & Technology, Abu Dhabi, UAE

**Keywords:** Engineering, Energy, Modelling, Economics

## Abstract

Maintaining reliable energy supplies with resilience to extreme weather, water shortage and rising electricity and cooling demand is crucial to successfully implementing the clean energy transition. The integrated power and water systems found in several hyper-arid countries, featuring cooling-driven electrical demand and near-total dependence on seawater desalination, offer case studies illustrating energy system robustness to these conditions. We use linear optimization to minimize costs in a model system based on the resiliency-oriented energy system of the United Arab Emirates (UAE) while progressively decarbonizing the energy mix. We demonstrate that high levels of renewable energy adoption are economically favorable under conservative future technology cost assumptions, even including strict resiliency requirements, and draw conclusions for other energy systems making the transition to renewable energy under challenging climatic conditions, especially regarding the role of water desalination, demand flexibility, energy storage, and suitability of conventional design rules for ensuring resilience in renewable-dominated systems.

## Highlights

• We optimize net-zero future energy systems for the United Arab Emirates
• Systems are designed to be resilient to demand spikes and low solar-output years
• Modeled future power generation costs are similar to current system
• Demand flexibility, energy storage and seawater desalination are critical to resilience

## Graphical abstract

**Figure:**
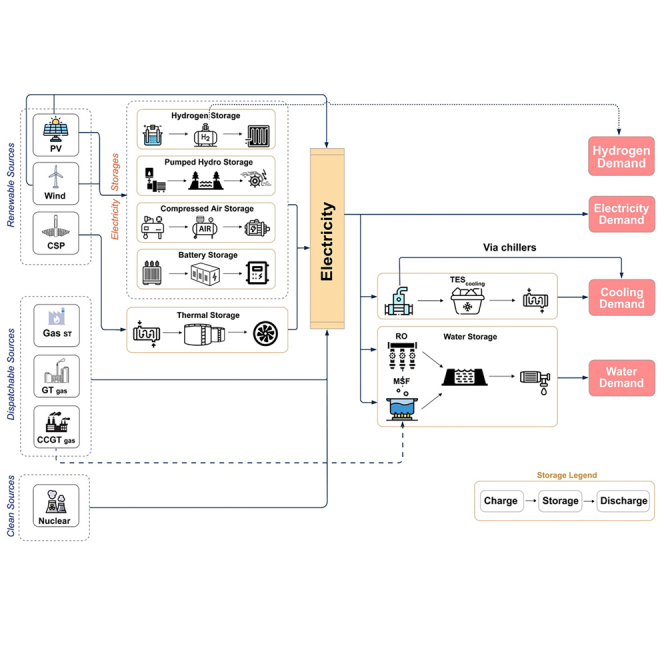

## Introduction

Over the past several years, countries around the world have made commitments to transition their energy systems from fossil fuel-based technologies to clean energy systems.^1^^,^^2^ A crucial question is how this transition can be carried out while maintaining appropriate reserves and safety factors.^3^^,^^4^^,^^5^^,^^6^ As renewable energy sources have become cost-competitive without subsidies^7^ and comprise a growing share of total generating capacity, the energy transition has reached a new phase in which renewables are no longer a niche “alternative” energy but a mainstream generation option with a critical role in maintaining an economical, secure, and sustainable electricity supply.^8^^,^^9^^,^^10^ At the same time, a changing climate^11^^,^^12^^,^^13^^,^^14^ and geopolitical conflicts^15^ have stressed energy systems and disrupted fossil fuel and electricity markets, leading to conventional energy technologies that are typically regarded as “firm” power sources becoming increasingly vulnerable to disruption, often when most needed.^16^ A successful energy transition must therefore identify opportunities to maintain and, where possible, enhance system availability, reliability, and resilience in the face of these challenges.

In this context, it is instructive to study the operation and evolution of energy systems that are designed for resilience under conditions of extreme heat and water scarcity. This paper identifies key lessons relevant to the resiliency of renewable energy systems based on the United Arab Emirates (UAE) current steps and modeled transition toward a net-zero energy system. For decades, the UAE has operated an integrated power and water system that has reliably serviced the needs of the country and its inhabitants despite a harsh natural environment. Unlike many systems in temperate climates, there is no assumption of water availability, whether for human, agricultural, or industrial use, or for the power generation process itself, and air conditioning is essential throughout the summer months when temperatures can exceed 50°C. Inland power stations are air-cooled whereas those near the coast are seawater cooled; most of these are co-generation or combined power & water (CPW) facilities, typically combined cycle gas turbine (CCGT) systems that utilize waste heat in thermal desalination units, which provided 85% of the country’s municipal freshwater in 2021 with the remaining 15% supplied by reverse osmosis (RO) plants.^17^^,^^18^^,^^19^ Several open-cycle gas turbine (GT) systems are also in operation, largely for peak load coverage and reserve capacity. In the Emirate of Dubai, ∼20% of cooling demand is supplied by district cooling plants,^20^ while conventional central and split air conditioning systems supply the remainder. Large reserve margins (RM) on all capacities are required to ensure stability of supply; in Dubai, for example, desalination capacity is sufficient to cover all municipal water demand in addition to 2 days of peak water demand as reserve stored in reservoirs, while power generation capacity is required to be 25% greater than peak demand.^17^ Power stations maintain emergency fuel reserves which can be used for power production and desalination in the event of any disruption to the natural gas supply.^21^

The success of this system in providing reliable electricity supplies can be quantified by the System Average Interruption Duration Index (SAIDI), an industry-standard metric of the average annual electricity outage duration per connected customer account.^22^ SAIDI was less than 2 min in Dubai in 2021,^17^ while on average US utilities had 138^23^ and European utilities ranged from 9 to 270 min.^24^ In 2021, the UAE committed to a target of net-zero greenhouse gas emissions by 2050,^25^ which has driven national utility companies to accelerate their planned transition to clean energy sources. Hence the UAE’s energy providers can be seen as operating under a “triple mandate” to maintain power and water supply that is simultaneously *reliable*, *affordable*, and *sustainable*. In this study, we treat the UAE as a model system to highlight considerations for the energy transition under conditions of extreme temperatures and water scarcity that must increasingly be taken into consideration in both developing and established energy and water systems around the world.

We conduct our study by implementing a linear optimization to minimize cost under constraints limiting emissions and enforcing power reserve margins consistent with those seen in the UAE and across the region, which has conventionally been seen as a critical component of system resilience. The use of linear optimization to study energy system evolution is well established in previous literature. Past studies on large-scale adoption of renewable energy have generally either focused on the energy system as a whole or the electricity system specifically. The former uses a broader but less detailed approach to estimate total transition requirements and installation rates.^26^^,^^27^^,^^28^^,^^29^ The latter primarily use detailed (hourly) modeling for individual countries^30^^,^^31^ and regions including trade^32^^,^^33^ and storage options using custom proprietary models. Previous research on the UAE investigated RE electricity shares of 50% by 2030 using an hourly simulation model^34^ and a 10% total share of primary energy by the same year using an aggregated energy model.^35^ An hourly optimization approach for 100% RE systems^2^^,^^36^ has been implemented across different global regions but does not capture country-specific details and context which can be instructive for drawing conclusions related to particular situations of interest for resilience—e.g., outlier weather events and unplanned disruptions.

Of particular relevance in our study is the impact of the transition to clean or renewable energy on overall system costs. Jenkins et al. indicated that the increase in system cost is non-linear against renewable adoption, with costs roughly doubling when the renewable fraction is increased from 20% to 80%^37^; however, these values are heavily dependent on the assumed costs of technologies and must be regularly updated in the rapidly evolving green energy sector. De Sisternes et al. demonstrated the importance of low cost energy storage for enabling cost-effective large scale renewable generation on a system level.^38^ Sepulveda et al. identified at least 100 h duration and less than $20/kWh_cap_ as the criteria for economically viable long-duration storage,^39^ while others have found values as low as $3/kWh _cap_ to be necessary at these durations.^40^ Hernandez and Gencer indicated that hydrogen provides the most cost-effective solution for this function^41^ assuming costs reported for underground hydrogen storage^42^; other authors have shared similar findings on the potential role of hydrogen in the energy system^43^ and as a sector-coupling vector^44^ Other studies have found compressed air energy storage and pumped hydropower to be favorable for medium- to long-term energy storage.^45^

**Figure 1:**
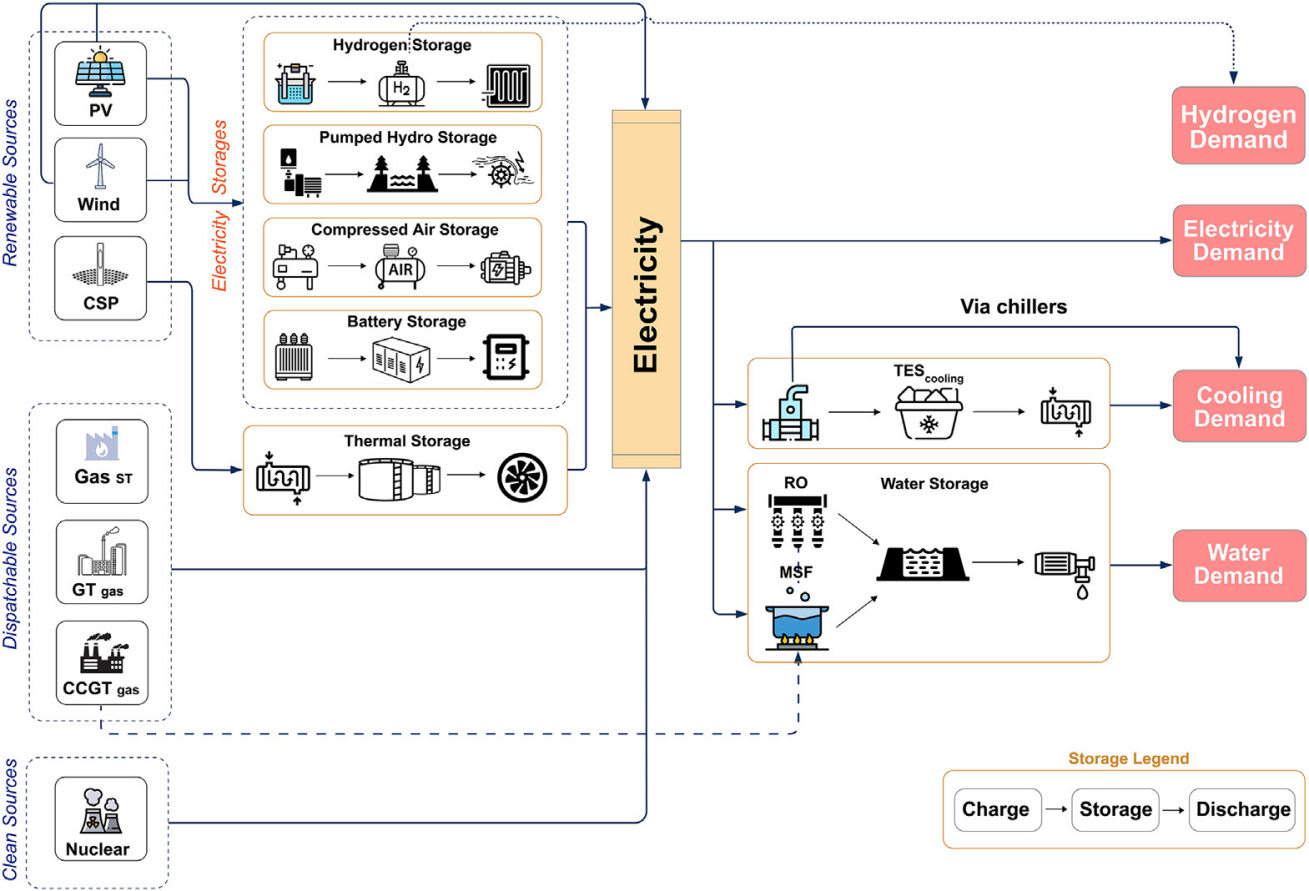
Illustration of model architecture involving various energy sources supplying storage systems to satisfy power, water, cooling and hydrogen demands

We model a multi-energy system^46^^,^^47^^,^^48^ meeting three independent needs, for water, cooling, and other electricity demand as has been done previously for other energy-water systems,^49^^,^^50^ but adding the aspect of water desalination as a central component of the energy system. To render the electricity/water system transformation with clarity, we assess the effect of hydrogen demand separately and discuss it comparatively in the results section. Based on a “menu” of generation and storage options (Figure 1) and associated costs (see LCOE and amortized cost assessment for different generators and storages section under STAR Methods), we implement the linear optimization in Python with the Gurobi optimizer. Power generation options include gas turbine (GT), steam turbine (ST), combined cycle (CCGT) and nuclear power stations, and ground-mounted photovoltaic, onshore wind, and concentrated solar power (CSP) renewable energy options; desalination options include multi-stage flash (MSF) thermal desalination coupled to CCGT plants and RO powered by electricity. Storage options include Li-ion batteries, pumped hydropower, solar thermal energy storage (linked to CSP plants), and adiabatic compressed air energy storage (ACAES); hydrogen storage which incorporates electrolyzers and fuel cells to produce and reconvert hydrogen from and to electricity; as well as reservoir storage for water and cold thermal storage for cooling. The hydrogen storage system is also responsible for meeting hydrogen demand, where applicable (details of all storage options shown in Figure 1).

Using the renewable generation and demand profiles (see renewable energy profiles), we ensure that supply meets demand at all hours as well as numerous other constraints representing the physical behavior and limitations of the different components of the energy system (see optimization model). Details of demand, resource, and cost calculations are provided in the methodology (see demand profiles creation, weather file creation, renewable energy profiles, and LCOE and amortized cost assessment for different generators and storages sections under STAR Methods). To ensure resilience to low-probability extreme conditions, we “stress test” the designed systems by forcing them to operate under the solar resource profile from the year with the lowest total solar irradiance (GHI) from a dataset of 15 years (see weather file creation and renewable energy profiles sections under STAR Methods). A system is considered resilient to historically observed conditions only if it meets power, water, and cooling demand at all hours under the three worst solar years in the database.

## Results and discussion

**Figure 2:**
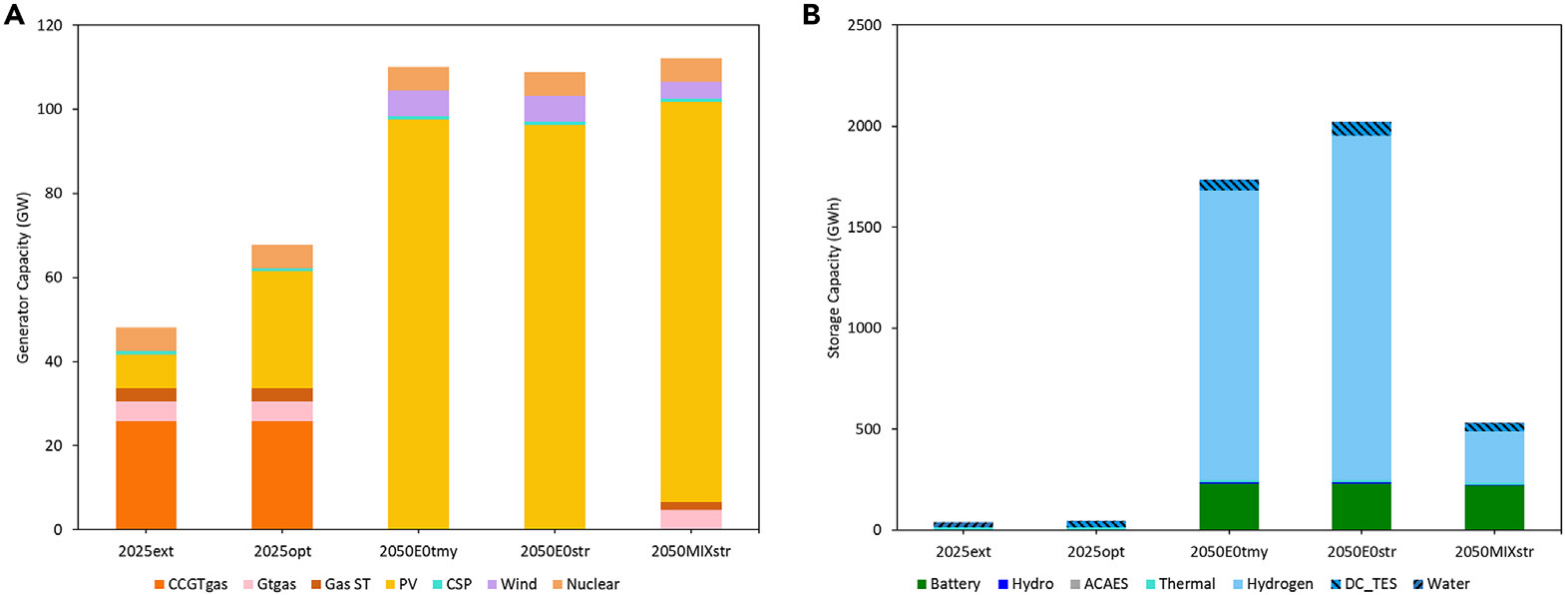
Generation and storage capacities for scenarios listed in Table 1 Optimization results showcasing capacities for (A) generators and (B) storages. Patterned storage graphs are non-electrical storages.

**Figure 3:**
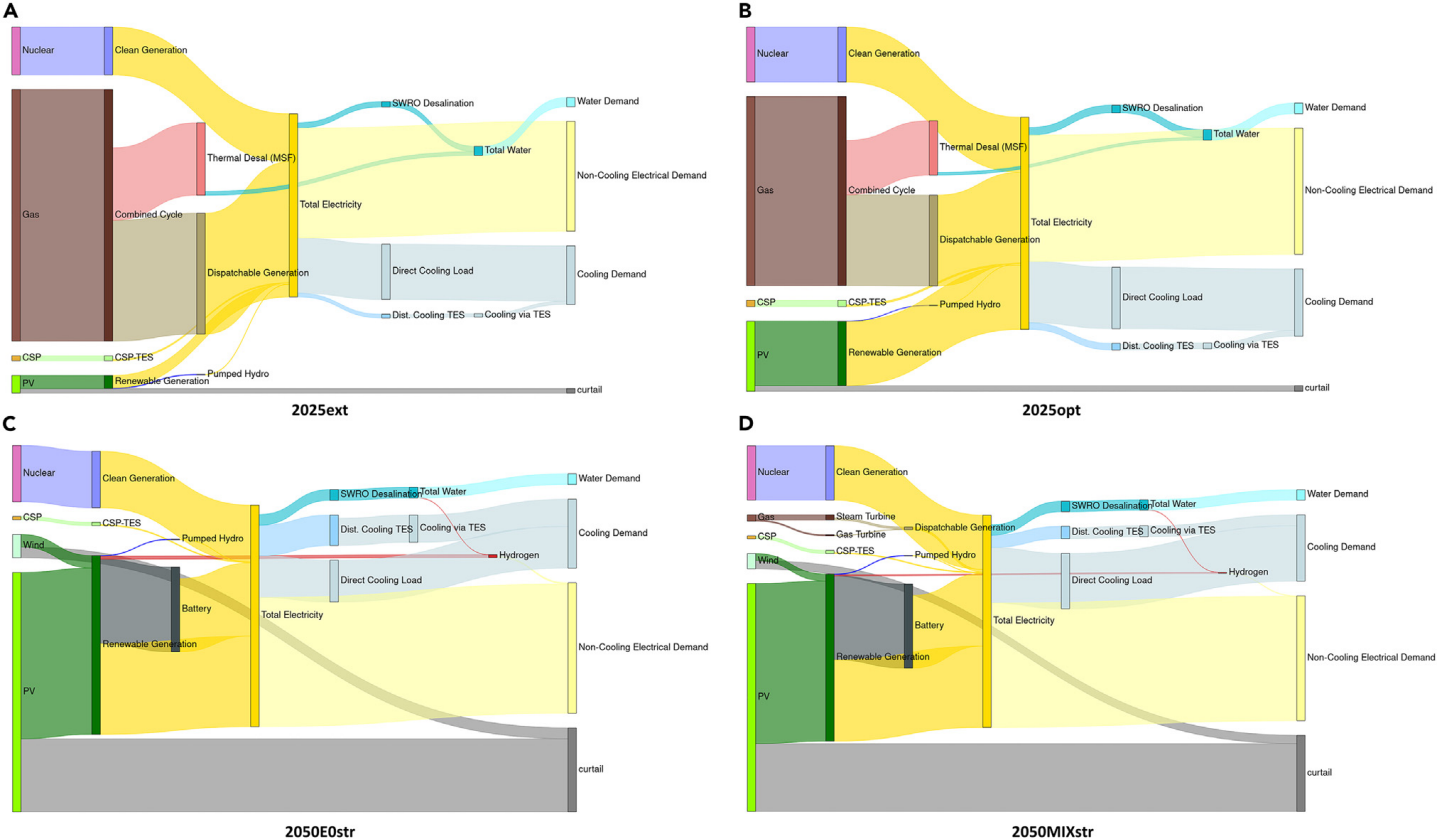
Annual energy flows from various energy sources to satisfy respective demands directly or via storages across scenarios (A) 2025ext, current demand, planned expansion, current costs, TMY; (B) 2025opt, current demand cost-optimal expansion, current costs, TMY; (C) 2050 demand, 100% clean energy, 2050 costs, stress year; (D) 2050 demand mixed system CO2 capture & carbon pricing, 2050 costs, stress year.

**Figure 4:**
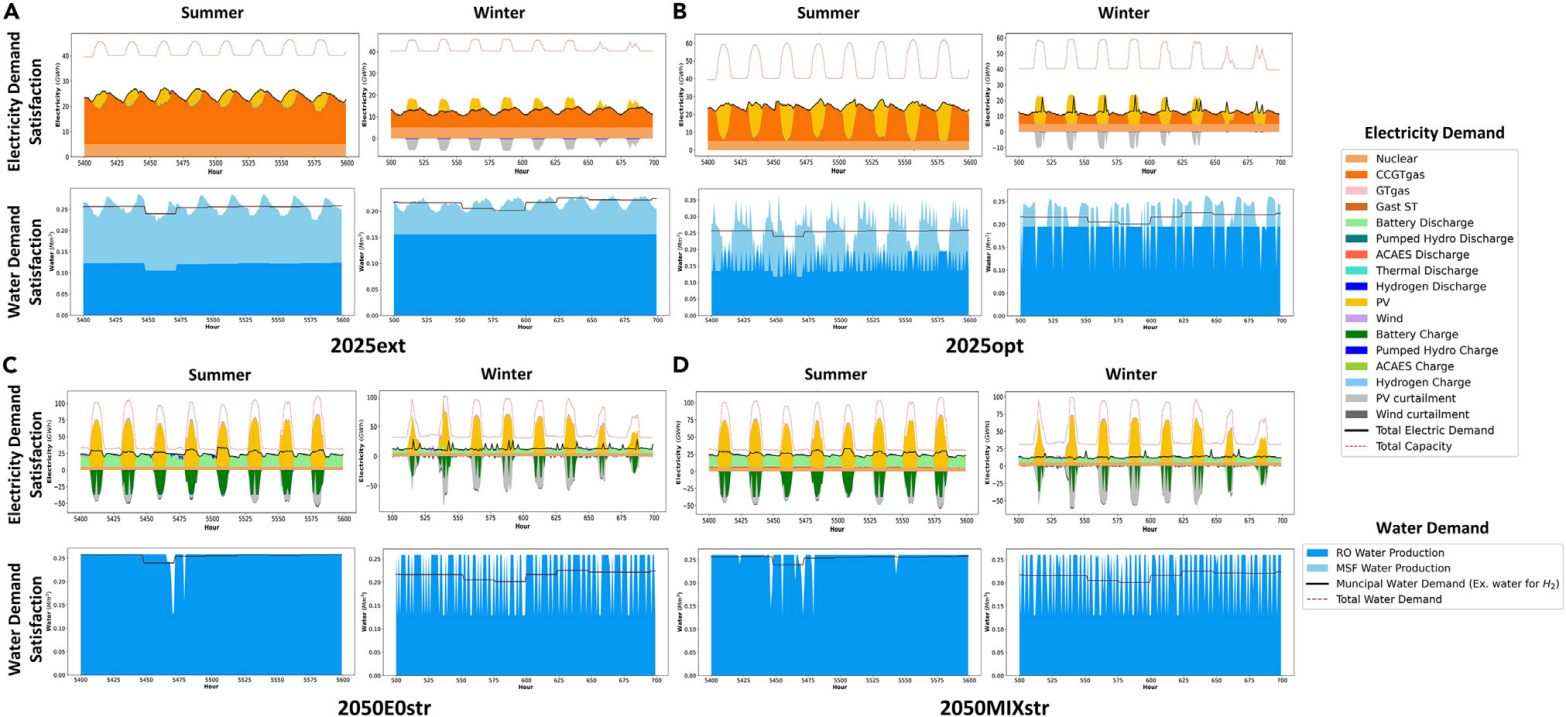
Electricity and water demand satisfaction figures in summer and winter across scenarios (A) 2025ext, current demand, planned expansion, current costs, TMY; (B) 2025opt, current demand cost-optimal expansion, current costs, TMY; (C) 2050 demand, 100% clean energy, 2050 costs, stress year; D) 2050 demand mixed system CO2 capture & carbon pricing, 2050 costs, stress year.

A total of 5 main scenarios are modeled, with results and brief descriptions listed in Table 1 (details of the inputs to each simulation are described in this section). As a benchmark, a system incorporating all existing and under-construction generating capacity in the three major UAE utilities (DEWA—Dubai Electricity & Water Authority, SEWA—Sharjah Electricity & Water Authority, and EWEC—Emirates Water & Electricity Corporation supplying Abu Dhabi and the Northern Emirates of UAE) is first modeled supplying estimated 2021 demand for electricity, cooling, and water, the most recent demand year available. The resulting configuration, i.e., capacities of all generators and storages, represents a business as usual configuration for the energy system by mid-decade, is listed in Table 1 and shown in Figure 2 as scenario 2025ext. Energy flow diagrams for the resulting system are displayed in Figure 3. The total capacity of thermal energy storage for district cooling (TES_cooling_) is limited to the currently available total capacity of district cooling in Dubai and Abu Dhabi, of approximately 3 million refrigeration tons (RT), and an assumption of 1.5 h of storage across all district cooling capacity (see cooling demand section under STAR Methods). CCGT operates in load following mode, supplementing the base load power of the nuclear plant, with PV curtailment in the winter (Figure 4A). This curtailment can be attributed to the fact that CCGT must operate in order to satisfy water demand via multi-stage flash thermal desalination (MSF) since the RO desalination capacity is still not sufficient to supply all water needs (Figure 4A lower panels). This behavior is typical of regional energy systems with coupled power & water production and has motivated the recent move toward increased use of RO, which can be driven by any electricity source and imposes no constraints on power system operation. 18 h of water storage are utilized (5.74Mm^3^ vs. 0.30 Mm^3^/h peak demand, the highest number of any of our modeled scenarios that is nonetheless far below the 48-h water storage requirement imposed by local regulations and satisfied by the major utilities (indicating that this requirement is more than sufficient to ensure resilience of the water supply to expected conditions). The overall cost of electricity is 52.4$/MWh_consumed_ and the total CO_2_ intensity is 261 kgCO_2_/MWh_consumed_.

**Table 1:** Optimized system configuration results across notable scenarios

SCENARIO		2025ext	2025opt	2050E0opt	2050E0str	2050MIXstr
Description		current demand, planned expansion, current costs, TMY	current demand cost-optimal expansion, current costs, TMY	2050 demand 100% clean energy, 2050 costs, TMY	2050 demand 100% clean energy, 2050 costs, Stress year	2050 demand Mixed system CO_2_ capture & carbon pricing 2050 costs, Stress Year
**a) Generation Capacities**						

*Renewables*	PV (GW)	8	27.8	97.6	96.4	95.2
	CSP (GW)	0.7	0.7	0.7	0.7	0.7
	Wind (GW)	0	0	6	6	4.1
*Gas*	CCGT (GW)	25.8	25.8	0	0	0
	GT (GW)	4.6	4.6	0	0	4.7
	Gas ST (GW)	3.3	3.3	0	0	1.8
*Nuclear*	Nuclear (GW)	5.6	5.6	5.6	5.6	5.6

**b) Storage Capacities**						

*Lithium Ion Battery*	Charge (GW)	0	0	39.6	36.2	36.9
	Storage (GWh_cap_)	0	0	230.4	229.5	219.2
	Discharge (GW)	0	0	22.1	20.5	18.2
*Pumped Hydro Storage (PHS)*	Charge (GW)	0.3	0.3	0.44	0.62	0.46
	Storage (GWh)	1.5	1.5	7.5	7.75	4
	Discharge (GW)	0.25	0.25	0.25	0.25	0.25
*Adiabatic Compressed Air Energy System (ACAES)*	Charge (GW)	0	0	0	0	0
	Storage (GWh)	0	0	0	0	0
	Discharge (GW)	0	0	0	0	0
*Thermal (CSP Molten Salts)*	Charge (GW_th_)	5.25	5.25	5.25	5.25	5.25
	Storage (GWh_th_)	31.5	31.5	31.5	31.5	31.5
	Discharge (GW)	0.7	0.7	0.85	0.8	0.72
*Chillers/Chilled Water Storage*	Charge (GW)	45.8	40.7	41.2	43.4	42.7
	Storage (GWh_th_)	16.9	87.9	138.7	181.5	110.9
	Discharge (GW_th_)	18	14.7	17.6	17.2	12.6
*Water Desalination*	Charge RO (Mm^3^h)	0.16	0.19	0.26	0.26	0.26
	Charge MSF (Mm^3^h)	0.26	0.26	0	0	0
	Storage (Mm^3^) [requirement for 48 h]	5.47 [14.4]	0.61 [15.4]	0.61 [12.9]	1.37 [12.9]	0.35 [12.9]
	Discharge (Mm^3^h)	0.30	0.32	0.27	0.27	0.27
*Hydrogen*	Charge (GW)	0	0	1.2	0.34	0.25
	Storage (GWh_th_)	0	0	1,004.1	1,194.4	178.5
	Discharge (GW)	0	0	5.6	8.84	0.75

**c) Financial Metrics**						

Total System Cost (billion $)		**10.9**	**10.4**	**9.5**	**9.8**	**9.3**
Cost of Electricity Consumed ($/MWh)		**52.4**	**49.3**	**48.1**	**49.1**	**47.5**
Total Curtailment (GWh)		4,145	4,548	74,822	65,845	61,907
Cost of Electricity Generation ($/MWh)		48.6	45.6	30.6	32.4	31.9

**d) CO**_**2**_**Metrics**						

CO_2_ emissions (tons/GWh generated)		242	157	0	0	1
CO_2_ emissions (tons/GWh consumed)		261	169	0	0	2

Sub tables displaying: (a) generation capacities, (b) storage technologies with charging, storage, and discharging capacities, and (c) financial and (d) CO2 metrics. Bold highlight the total system cost and the cost of electricity consumed across the scenarios.

**Figure 5:**
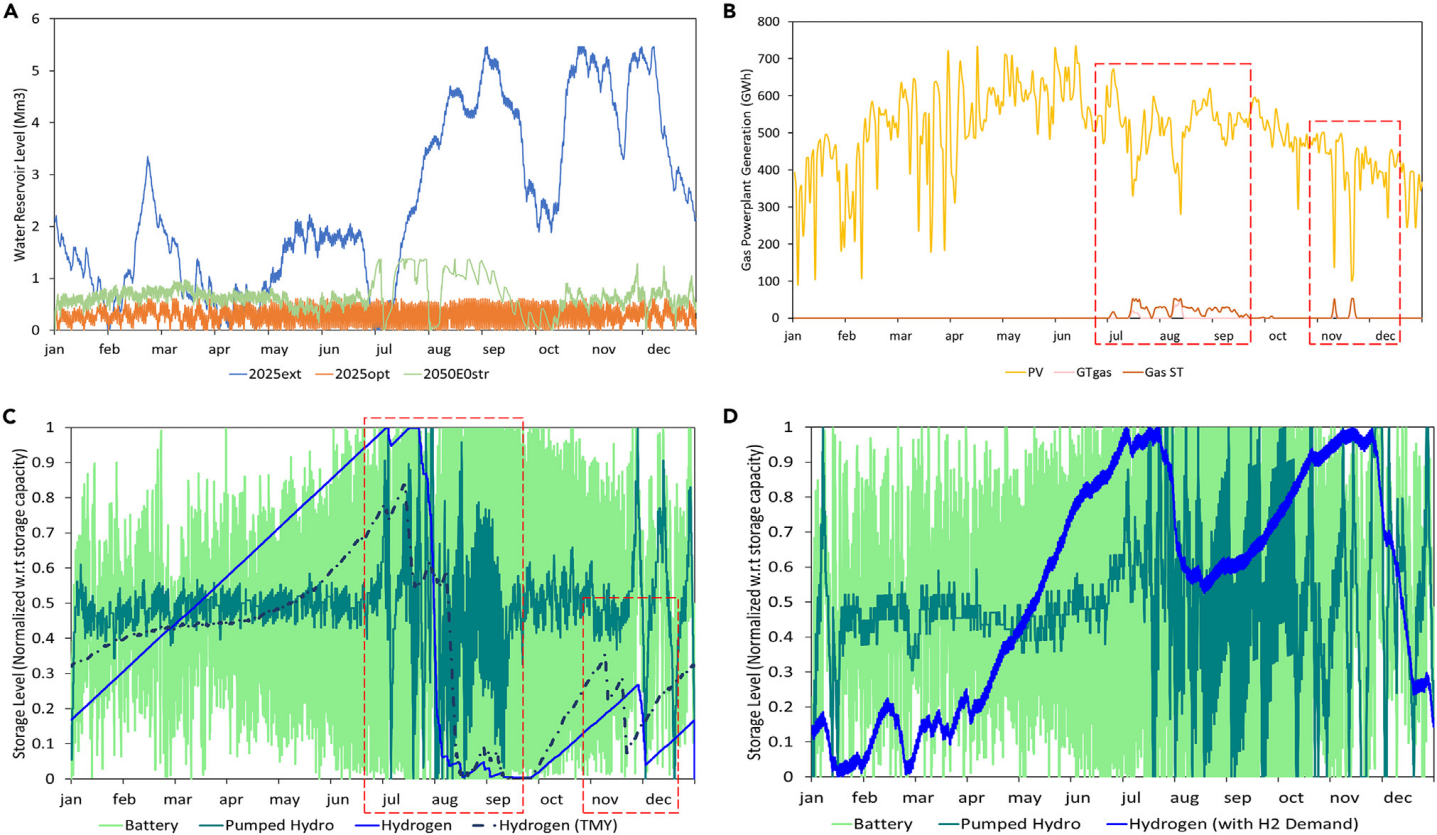
Storage levels over the year for energy storage systems showing the use of different technologies for different durations of storage (A) Water reservoir levels across scenarios. (B) Operation of gas power plants during low PV production showing energy shifts in scenario 2050MIXstr outlined in Table 1. (C) Use of energy storage systems in 2050MIXstr (battery, pumped hydro, and hydrogen) to provide daily, multi-day and seasonal storage/power backup, as well as the lower utilization of seasonal hydrogen storage when the same system is operated under TMY conditions. (D) Interplay of various storages as daily, weekly, and seasonal cycles for future RE and nuclear scenario with green H2 demand. The red boxes in (B) and (C) show how the drop in PV production leads to sudden discharge of hydrogen storage to meet the electricity demand.

We then re-optimize the system allowing additional capacity to be built in addition to what already exists, representing a cost-optimized near-future energy system (Scenario 2025opt). Three noteworthy features are seen in this newly modeled system: an increase in solar capacity from 8 to 28 GW; an increase in thermal storage for cooling from 17 to 88 GWh_th_; and an increase in RO capacity from 0.16 to 0.2 Mm^3^. The increased RO capacity avoids the need to operate CCGT to provide a waste heat resource for thermal desalination and reduces the water reservoir capacity that must be used to balance seasonal variations in water production (Figure 5A); less than 2 h of water storage is utilized (0.61Mm^3^ vs. 0.32 Mm^3^/h) peak demand. The additional thermal storage increases the flexibility of the cooling load, allowing more of this load to be shifted into the solar generation peak. This flexibility, in turn, allows more PV to be used without the need for electrical energy storage, as is seen from the greatly increased contribution of PV in Figure 3B and as excess generation can be directly consumed by chillers. The total CO_2_ emissions per MWh consumed drops to 169 kg/MWh and the cost falls to 49.3 $/MWh due to the increased use of PV. Under stress testing, both systems are resilient to the worst 3 solar years (see S1.1).

We then consider future systems based on projected 2050 technology costs (see LCOE and amortized cost assessment for different generators and storages section under STAR Methods). In keeping with the UAE’s net-zero target and the overall purpose of this study, we constrain the energy system in 2050 to net-zero CO_2_ emissions (Scenario 2050E0opt). To realize this, we first test a cost-optimized system that utilizes no gas-fired capacity. Existing nuclear, CSP, and pumped hydropower capacity is retained as these systems will still be within their service life in 2050. The remaining energy demand is met by 97.6 GW of PV and 6GW of wind at a total cost of 48.1 $/MWh. Batteries are heavily used (∼10.5 h with 22 GW discharge capacity) in addition to hydrogen providing seasonal energy storage; cooling storage, and water reservoir capacities increase further. The same power reserve margins are required as in Scenarios 2025ext and 2025opt; however, this majority-renewable system is incapable of meeting demand in adverse solar years when stress-tested (failing to meet demand during 9 h over the year).

To improve resilience, we consider two options. In the first, we re-optimize the system to meet demand in the worst solar year in the database (Scenario 2050E0str); this system configuration increases 3.4% pumped hydro storage capacity and 18.9% hydrogen storage capacity using excess RE generation to charge these storages instead of curtailing. This “overbuilt” system is able to meet demand in the second and third worst solar years (see S1.2), with a cost increase to 49.1 $/MWh due to the change in capacities as shown in Table 1. In the second approach, we allow gas-fired capacity to be built with CO_2_ emissions accounted for in two ways: first, by the option of gas-fired plants with point-CO_2_ capture; and second, by the application of a high CO_2_ emissions price of 1000 $/ton, based on recent cost estimates for direct air capture for atmospheric CO_2_ removal^51^ (Scenario 2050MIXstr). In terms of the overall energy contribution of different sources, the difference between this system and Scenario 3 is fairly small as seen from the annual energy flows in Figures 3C and 3D. The gas-fired capacity operates during periods of high demand and when solar output is abnormally low (Figure 5B). This system achieves a lower cost of 47.5 $/MWh with resilience to all tested conditions, with the caveat that a total of 1ton/GWh/year of CO_2_ is produced, which must be permanently sequestered to satisfy the requirement of net-zero CO_2_ emissions.

When describing the cost of energy ($/MWh) in future energy systems (Scenarios 2050E0opt, 2050E0str, 2050MIXstr**)**, the primary source of uncertainty in these estimates comes from the uncertainty in future demand or prices of fuel and technologies; in the additional scenarios referenced in the discussion and detailed in the supplementary material show variations of 5–10% in total electricity cost under large variations of storage capex. However, the manuscript is primarily concerned with showing the relative difference between scenarios with different sets of constraints (e.g., true-zero vs. net-zero emissions systems) assuming a particular set of costs that is consistent across the different scenarios being compared.

Several design features have made the historical UAE energy system resilient to the harsh conditions prevalent in the country and region, namely freshwater scarcity and daytime temperatures of 40°C–50°C throughout the summer.^52^ These conditions are addressed by the use of desalination capacity covering all municipal and partially agricultural needs, and by sizing electrical generation capacity to a cooling-driven summer demand peak with high power reserve margins. These options are available to any power system with access to adequate energy supplies, an open coastline, and access to financing to construct the needed capacity; the cost of power and water production in this system can be covered by tariffs consistent with global norms, as are observed in the UAE.^53^ Our results also show that the entire municipal water demand of the UAE can be met by RO consuming approximately 5% of total electricity, indicating that desalination is sufficiently energy-efficient to make a meaningful contribution to a sustainable energy-water system (when appropriate environmental precautions are taken).

Furthermore, the results of future energy system optimizations can maintain or reduce current generation costs with resilience. Even when the energy system is built to withstand historically observed adverse renewable resource conditions (e.g., long cloudy spells) and transition completely to zero-emissions energy sources. This shows that, under modest assumptions regarding future technology cost reductions, energy systems can be developed that satisfy the “triple mandate” of affordability, availability, and sustainability even under conditions that would be regarded as extreme in most areas of the world. We identify three points that are critical to maintaining resilience and warrant further discussion: load flexibility, appropriate deployment of energy storage, and the need for adequate design rules to govern the construction of resilient clean energy systems.

### Energy storage

In our optimization results, we see the deployment of different storage technologies for different applications and on different time scales. Under current technology costs (see Scenarios 2025ext, 2025opt, Tables 1 and S1.3), minimal energy storage is used. The most cost-effective configurations simply utilize renewable energy when it is available (curtailing excess) and use gas to meet the remaining demand, while load flexibility is used to maximize the overlap between RE generation and demand. However, under the 2050 cost structure, multiple forms of energy storage are used. Scenario 2050E0str (worst-year, 0-emissions system) illustrates the differing roles of different technologies (storage energy and power capacities listed in Table 1). Batteries are used for daily cycling while PHS operates in a “mixed” mode where part of the capacity undergoes daily cycling and part stores energy over longer durations for discharge in periods of low RE production, and hydrogen provides seasonal storage as seen in Figure 5C. In Scenario 2050E0str, hydrogen capacity is sufficient to cover large net demand spikes caused by periods of low solar production during the summer; when the same system is run using the solar generation of the typical year, the hydrogen storage is not fully utilized as shown by the dotted line in Figure 5C. This illustrates the importance of building for adverse conditions; the system design for typical conditions (Scenario 2050E0opt) would not have been resilient to the abnormally low summer solar generation observed in the worst year. We note how storage technologies “sort” into different use cases based on the relative costs of power & storage components, where batteries, with high storage cost and low power costs, are used for shorter-duration storage; hydrogen, with high power costs and low storage costs, is used for long-duration storage; and CSP and PHS, with intermediate costs in both categories, are used for medium duration or mixed-mode storage as observed. This sorting based on storage technology cost structure is observed generally in studies of future energy systems around the world^54^^,^^55^^,^^56^ with some variation based on the technologies available; for example in a study on the European market,^57^ hydrogen covered multi-day storage requirements and reservoir hydro covered seasonal variability, while in our study reservoir hydro is not an option and hydrogen is used for seasonal storage.

The operating mode of different storage technologies is strongly dependent on the relative costs of the power and energy components of different technologies relative to each other. Scenario 2050E0str assumes battery storage costs of 62 $/kWh in 2050. If instead, we maintain the present-day battery storage cost assumption of 220 $/kWh, no battery capacity is deployed (Scenario 2050E0str.2) (see S1.3). Instead, CSP, PHS, and ACAES (which appears viable based on costs reported in the literature but require further investigation to determine whether these costs can actually be realized in the region under study) are used for daily cycling, while hydrogen provides seasonal storage and a small amount of daily cycling. This reflects the current situation where batteries are still not widely considered economically viable for medium-duration applications (>4 h) on an unsubsidized cost basis alone. When an intermediate cost for batteries (140 $/kWh) is used, lower-capacity battery systems are built and utilized only for short “bursts” of power of 1–2 h, while ACAES, PHS, and CSP undergo daily cycling and hydrogen again provides seasonal storage (Scenario 2050E0str.3) (see S1.3). With this analysis, we showcase how energy storage technologies can be arranged as a “ladder,” with short-duration technologies prioritizing low power costs at the bottom, and long duration technologies prioritizing low storage costs at the top, and also how the boundaries between the different storage regimes (the “rungs” of the ladder) are impacted by the relative costs of the different technology options.

On the “top” of the ladder, there is a decision to be made about how to deal with seasonal variations—seasonal energy storage or overbuilding RE and curtailing during the low demand period. We demonstrate how this is also determined by the cost of seasonal storage technologies (hydrogen in our model). In scenario 2050MIXstr, under the projected H_2_ costs of 300$/kW electrolyzer, 1.5$/kWh cavern storage, and 800$/kW fuel cell, a mixed system with ∼5GW/1000GWh of H_2_ storage as well as ∼35% curtailment on renewable output is built in the cost optimized case (the use of cavern storage, while not definitively proven viable for the UAE, appears at least promising based on previous studies in the region^58^ and analyses of depleted gas reservoirs as H_2_ storage facilities^59^). If a higher price of 650$/kW electrolyzer, 3.5 $/kWh cavern storage, and 1150 $/kW fuel cell is assumed, very little seasonal storage is used – only 0.5GW of H_2_, supplemented by 0.25GW pumped hydro with ∼40-h storage (Scenario 2050E0str.4) (see S1.3). ∼18% more PV capacity is built with greater curtailment in the winter. If, on the other hand, we further reduce electrolyzer and fuel cell costs, to 150 $/kW & 400 $/kW, respectively, the total curtailment is cut in half and most of the seasonal variation is covered by seasonal storage (Scenario 2050E0str.5) (see S1.3). Hence the specific technology choices for different energy storage needs, and even major system design choices such as whether to utilize seasonal storage, will be heavily dependent on the actual trajectory of technology costs over the coming decades.

### Load flexibility

From our analysis, the ability to shape the demand profile in response to energy availability emerges as a critical enabler of the expansion of renewable energy capacity in the near term at low cost. By avoiding the need for electrical energy storage, load flexibility allows system operators to take advantage of the low cost of generation from solar and wind systems, while minimizing the need for high-cost storage systems. In our study, the largest sources of load flexibility are thermal storage for cooling, and SWRO desalination. To quantify the value provided by flexibility through cooling storage, we optimized a system (Scenario 2025opt.1) (see S1.3) fixing TES_cooling_ capacity at the level of Scenario 2025ext (17 GWh_th_), but allowing other capacities to optimize as in Scenario 2025opt. Comparing Scenarios 2025opt and 2025opt.1, the former (TES_cooling_ capacity = 88GWh_th_) had a lower cost of electricity by 0.6 $/MWh, or a savings of 1.34 m$/GWh_th_ when flexibility was introduced. In the future, we anticipate that other loads such as electric vehicle charging, green hydrogen production, and building-level interventions will provide significant additional flexibility to the electricity demand. To quantitatively investigate the potentially largest of these, green hydrogen production, we model a modification of scenario 2050E0str (Scenario 2050E0str.1) (see S1.3) in which there is a demand for green hydrogen of 3.65 million metric tons/year, assumed to be evenly distributed across all hours, based on the industrial sector demand analysis by Gandhi et al.^60^ The cost of electricity production is 31 $/MWh_generated_ and 39 $/MWh_consumed_. By using renewable generation that would otherwise be curtailed (resulting in the daily cycle of hydrogen production seen in Figure 5D), the flexibility of hydrogen reduces the cost per MWh consumed by 25% relative to Scenario 2050MIXstr where hydrogen demand was not included. In all flexibility sources, some limitations apply in the real world. For example, thermal storage can only be cost-effectively deployed, at present, in district cooling systems; SWRO must maintain a minimum flow rate in order to avoid membrane fouling; and hydrogen production systems must be sufficiently utilized to cover system costs through hydrogen sales. A variety of metrics have been developed to quantify demand flexibility; in future work, we plan to address the quantification of flexibility from the perspective of energy system design in more detail.

### Design rules for resilient clean energy systems

One of the main legacy design rules typically used to ensure resilience of conventional energy systems, the power reserve margin, is not sufficient to guarantee resilience of renewable-dominated energy systems. This is illustrated by the fact that conventional or hybrid systems, when designed with a large power reserve margin, can supply sufficient power even when net demand is significantly higher than anticipated, but renewable-dominated systems cannot due to the depletion of energy storage systems. This highlights a critical difference between conventional systems, where availability of energy in the form of fuel is assumed and generating capacity, i.e., power plants, are sized only to deliver sufficient power at all times, and renewable-based systems, where energy is collected over time from the solar or wind resource, and the system must also be able to collect and store sufficient energy to meet instantaneous power demand at all times. Based on this critical difference, we can consider conventional systems as “power-denominated” systems, and renewable-based systems as “energy-denominated.” In a power-denominated system, adequate power reserve margins are sufficient to guarantee resilience as long as fuel supplies are not disrupted. In an energy-denominated system, the ability to quickly deliver additional power is not sufficient to guarantee resilience if energy stores are not adequately charged. This implies that an additional design rule is required to ensure a resilient energy-denominated system—an “energy reserve margin” which ensures that there will always be sufficient energy in storage to maintain supply to customers in the face of adverse conditions or unexpected events. This energy reserve would serve the same function as an on-site fuel reserve in a conventional power station, or a strategic fuel reserve at the national level, but incorporating this reserve into a clean energy system is non-trivial because it involves not only including sufficient energy storage capacity but ensuring that this capacity is adequately charged and is available when needed even under infrequent circumstances. Hence the proposed energy reserve margin would be a general metric incorporating both conventional fuel reserves and stored energy that would constrain both the design and operation of the energy system so that total energy reserves never fall below a specified level. For an initial assessment of this value, we can consider the difference between the energy storage capacities in our 3^rd^ and 4^th^ main scenarios, 2050E0opt and 2050E0str. 2050E0str, built for the worst solar year, is distinguished primarily by the inclusion of an additional 190GWh/3.2GW of long-duration storage (hydrogen). For the purposes of this study, we can consider 190GWh as the energy reserve margin required to maintain resilience to the worst year in a system built for the typical year. However, more detailed study to identify the most appropriate metric for quantifying this energy reserve, and suitable design strategies for incorporating it into future energy systems while incorporating the trade-offs between resilience and cost, is a critical subject for future work.

### Global insights from the UAE energy system

The scenarios described in this manuscript detail the ways that the UAE energy system currently satisfies, and can continue to satisfy in the future, the triple mandate of reliability, affordability, and sustainability, despite facing environmental conditions that have severely challenge energy systems in other parts of the world. Based on this we can consider ways to address a variety of issues impacting global energy systems, reducing environmental impact while improving the resilience of energy systems to the challenges that they increasingly face (Table 2). The specifics of these situations vary significantly, and our study is not intended to provide a detailed guide to avoiding energy shortages in all of them. Rather we seek to identify energy system characteristics that can enhance resilience across a range of different scenarios. Common features include (1) using renewable energy sources that are not dependent on fuel imports; (2) minimizing dependence on naturally occurring resources such as water whose long-term available is uncertain in many parts of the world; (3) including overcapacity and redundancy in system design; and (4) explicitly designing systems for the worst anticipated conditions.

**Table 2:** Major challenges faced by global energy systems and potential solutions

Energy system challenge	Examples	Solution in current/future UAE energy system	Relevant scenarios
Energy price instability	EU/UK heating/electricity price spikes due to geopolitical conflict & gas supply disruption^61^^,^^62^	Increased use of renewable & nuclear power that is independent of fossil fuel markets Regulated energy markets & system design based on built on long-term planning that insulate against market fluctuations.	2025opt 2050E0str 2050MIXstr all
Electricity shortage	Blackouts in Pakistan & parts of India due to elevated energy demand^63^^,^^64^ Extreme fuel shortage & grid collapse in Lebanon, Bangladesh^65^ “Load shedding” due to inadequate generation capacity in South Africa^66^	Size capacities for worst-case demand scenarios with adequate reserve margins. Increase the use of renewable and nuclear energy sources that do not require a continuous fuel supply; oversize storage capacities to maintain energy reserves. Adequately size generation capacities; increase flexibility of demand to avoid peaks exceeding available energy/capacity.	All 2050E0str 2025opt 2050E0str 2050E0str.1 2050MIXstr
Water shortage impacting energy production	Southern Europe & Western US freshwater scarcity^67^	Move toward seawater desalination rather than overuse of freshwater resources. Reduce dependency on power generation using natural water supplies and increase RE generation.	All
Grid failure during extreme events	Texas blizzard power failure due to unavailable reserve capacity, inadequate weatherization, and no connection to the national grid for backup. Blackouts in many countries due to high cooling demand during extreme heat waves^68^	Design for rare weather events. Allowing for diverse portfolio. Interconnections between neighboring regions/states that can support by providing power during emergencies.	2050E0str 2050MIXstr All (integrated system model vs. multiple independent utilities)

### Conclusions

In this study, we have considered the task of building energy systems that withstand the challenges posed by hyper-arid conditions, in particular life-threatening high temperatures and extreme water scarcity while being simultaneously resilient, clean, and cost-effective—the “triple mandate” of future energy system design. Using the integrated power-water-cooling system of the UAE as a model, we have shown that, in the near term, generation costs and emissions can be simultaneously reduced by combining increased deployment of renewable energy systems with measures to increase the flexibility of electricity demand. To achieve a net-zero emissions system by 2050, we find that declining costs of energy storage technologies will enable energy systems for typical historical conditions to be built at comparable cost per MWh consumed as today’s systems, and at lower cost if curtailed solar and wind energy can be utilized. We highlight water desalination as an energy and cost-effective solution to water scarcity with the potential to relieve water stress in many areas of the world if widely implemented with appropriate environmental safeguards. We furthermore identify core concepts that will be critical to the design of resilient clean energy systems, including load flexibility; appropriate selection of energy storage technologies conceptualized as a “ladder” ranging from short- to long-duration storage applications and requiring different cost structures and performance characteristics; and the need for new energy-denominated design safety parameters in addition to the conventional power-denominated reserve margin and fuel reserve requirements. As global energy systems undergo rapid transformation toward net-zero configurations, it is essential that maintaining and enhancing resilience be a central goal of energy system design. We present the work with the aim of drawing lessons from a national energy system that has achieved resilience and reliability under globally extreme environmental conditions that can inform the development of resilient renewable energy systems around the world.

### Limitations of the study

This study optimizes energy systems over a single design year at hourly resolution based on fixed input profiles of demand and RE generation. Therefore it does not consider inter-year demand variations e.g., changes in cooling load due to weather or sub-hourly phenomena such as PV output variations due to passing clouds. Furthermore as the capacities of all generators of each type are aggregated into a single parameter, differences in cost or performance between old and new capacity are not explicitly treated. These aspects will be investigated more thoroughly in future work.

## STAR★Methods

### Key resources table

**Table:** 

REAGENT or RESOURCE	SOURCE	IDENTIFIER
**Software and algorithms**		

Gurobi Optimizer	Gurobi Optimization^69^	https://www.gurobi.com
Python	-	-
System Advisor Model (SAM)	National Renewable Energy Laboratory ^70^	https://sam.nrel.gov/

### Resources availability

#### Lead contact

Further information and requests should be directed to the lead contact and corresponding author, Harry Apostoleris (harry.nicholas@dewa.gov.ae; harry.apostoleris@gmail.com).

#### Materials availability

This study did not generate new unique materials.

#### Data and code availability

• Majority of the useful data used in this study is included in the supplemental information.
• The original code can be available on request with the lead contact.
• Any additional information required to reanalyze the data reported in this paper is available from the lead contact upon request.

### Method details

#### Demand profiles creation

In order to model the multi-energy system, we separately specify the electricity, cooling, and water loads. We also consider an independent demand profile for hydrogen to satisfy industrial needs for float glass, ammonia, and steel production, the latter two being significant fossil fuel consumers and exported commodities.

Figure below shows the publicly available monthly energy demand profiles over several years for the emirate of Dubai (accessed from Dubai Pulse).

**Figure:**
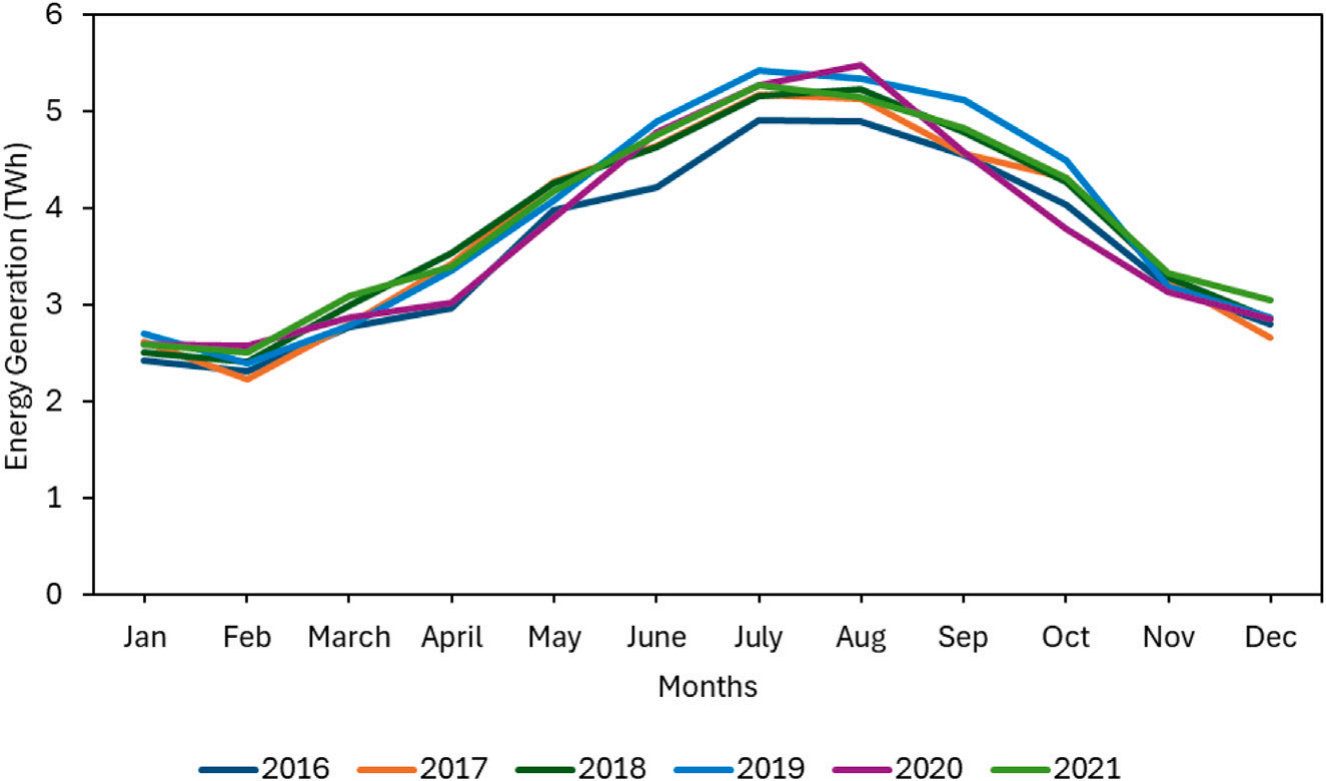

Monthly Energy Production in Dubai from 2016 to 2021 (accessed from Dubai Pulse).

#### Cooling demand

Given that no actual cooling data at the country level are available, we synthesize such a time series in order to investigate the possibility of handling the cooling demand separately and allowing specialized storage to cater to it. We take detailed hourly simulations of five representative buildings in the UAE (office building, commercial mall, detached housing, apartment building, and hotel) for an entire year using an open source building energy modeling package (EnergyPlus). Each category is weighted to represent a relative contribution based on representative building footprint area and normalized. The cooling load is then scaled to be equal to 70% of the total electric load at the peak day based on prior estimates.^71^^,^^72^ This is then converted into thermal (cooling) energy equivalent assuming the average coefficient of performance (COP) of the residential chillers to be 2.8.^72^^,^^73^ A part of this cooling demand is met by district cooling facilities, which operate centralized cooling plants using large industrial chillers typical capacity ∼2500 refrigeration tons (RT)^74^^,^^75^) and thermal storage tanks with an approximated capacity of 1.5GWh_th_ of storage per GW_th_ of capacity,^76^ which circulate chilled water through pipes to the air conditioning systems of adjacent buildings.

#### Non-cooling electric demand

The base hourly electricity demand time series is the sum of Abu Dhabi and Dubai electricity demand from 2015.^77^ This time series is scaled so that total consumption matches the actual UAE consumption in 2021^17^^,^^18^^,^^19^ (the last year for which consumption data are available at the time of writing) under the assumption that the general patterns of energy use among the remaining Emirates would not be markedly different. Notably, it offers a more realistic representation than the only available fully synthesized time series. The cooling load is subtracted from this. This gives us a more realistic daily varying non-cooling electrical demand and a seasonally varying cooling demand.

#### Water demand

The water desalination load is based on the daily dispatch schedule of Abu Dhabi 2015^77^ and scaled according to the actual water consumption in 2021.^17^^,^^18^^,^^19^ We treat the daily load as spread equitably over the 24-h duration, with flexibility in desalination plant operation provided by water storage. The lesser seasonality of water demand in comparison to electricity demand is one of the factors behind the UAE’s move to expand its capacity of seawater reverse osmosis (RO) desalination systems,^78^ which can be operated as needed rather than being coupled to a specific power plant as is the case with existing thermal desalination capacity. The dips are a weekly occurrence corresponding with reduced water consumption over the weekends. This may be a result of using a combination of only Dubai and Abu Dhabi as representative of the entire country, as Dubai during the weekdays hosts many commuters from other parts of the country.

Together, these components can be seen as forming an integrated power-water-cooling system.

#### Weather file creation

The TMY file is created from a 15-year weather dataset (2006–2020) from Copernicus Atmosphere Monitoring Service (CAMS)^79^ for the location near MBR Solar Park, Dubai, UAE using the steps mentioned below:

a) Calculate the daily average for all the months in the 15-year dataset.
b) Find the median (P50) of each month in the 15-year dataset.
c) Select the month that has the closest daily average value w.r.t the median (P50) to create the TMY weather file.

The three worst years for resiliency analysis are selected based on the cumulative annual global horizontal irradiance (GHI) for the different years of the 15-year dataset. Figure below shows the GHI profiles for the different years of study.

**Figure:**
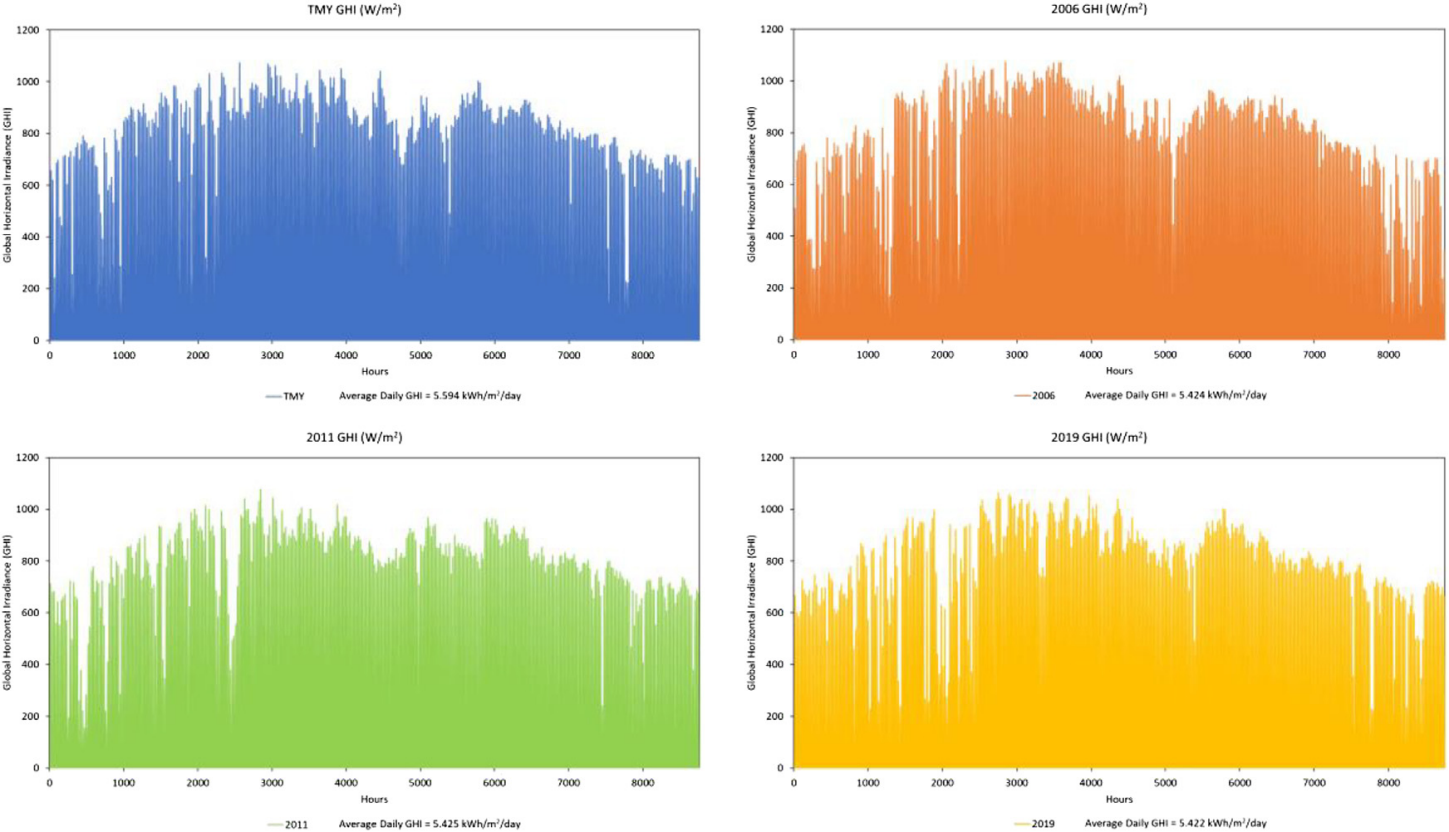

GHI profiles for TMY and three worst years (2006, 2011, 2019) used in the study.

#### Renewable energy profiles

The country’s primary renewable resource is solar energy^35^; most capacity is located in the central part of the country, where the strongest solar resource can be found.^80^^,^^81^ Horizontal single-axis tracking (HSAT) PV is preferred in recent projects as it has a higher energy yield when compared to fixed tilt PV in desert environments.^82^ Concentrated solar power (CSP) with thermal energy storage (TES) has also been pursued as a solution for low-cost overnight solar energy storage.^83^ Typical PV and CSP generation profiles have limited seasonal variation due to the relatively small differences in daily insolation from winter to summer at low latitudes. Some reduction in output during the summer is seen due to higher aerosol levels attenuating sunlight and extreme temperatures (with module temperatures exceeding 70°C) impacting PV module efficiency. Overall, the potential solar energy resource far exceeds the current energy demand in the country, as 5% covering of the total surface area with photovoltaic plants would imply a potential of over 400GW generating nearly 1000TWh/year at the current standard land-use efficiency of ∼100 MW/km^2^. While less prominent than solar, some wind resources exist in the mountainous north and east of the country^84^ and can partially compensate for the diurnal variation of solar. With a deployment density of 0.5 MWp/km^2^,^85^ the potential installed capacity could be roughly 6 GW.

We assessed the solar technologies’ power output profile using the weather files for the location near the Mohammed bin Rashid Al Maktoum solar park (24.8401, 55.3516), Dubai, UAE; from the open-source database Copernicus Atmosphere Monitoring Service (CAMS).^79^ East-west tracking c-Si photovoltaics and parabolic trough CSP were modeled in System Advisor Model (SAM) (v2021.12.2)^70^ as the dominant configuration for PV and CSP technologies, respectively, in the country and region.^86^ For horizontal single-axis tracking (HSAT) PV comprising of 1MW DC capacity and 1:1 AC to DC inverter ratio, the hourly AC electrical generation is given in [kWh/kW]; for parabolic trough CSP without thermal energy storage (TES), the thermal output for the collector array is given in [kWh_th_/kW_th_]. Wind generation profiles were estimated using a wind TMY for the hourly speed profile of a single year at 150m hub height (H) for two representative sites, one in the Western and one in the Northern region. The surface-area weighted average of the two installations was used as input for the SAM wind power simulation. Detailed solar (PV and CSP) and wind simulation parameters along with existing and planned plant locations are indicated in methodology (see weather file creation section under STAR Methods) and (see renewable energy profiles Section under STAR Methods) respectively.

The generation profiles (refer to main text Figures 1D–1F) of the three renewable energy technologies: PV, CSP, and Wind were from the System Advisor Model (SAM 2021.12.2) using GHI (see weather file creation section under STAR Methods) and wind speed profile (shown below), and a specific set of simulation parameters as shown in Table below. The wind speed data was obtained from satellite data (PVGIS) at a hub height (H) of 80m. Recent reports in the UAE show that studies are done at a hub height of 150m. Using wind profile power law:

$\frac{u}{u_{r}}={\left(\frac{z}{z_{r}}\right)}^{\alpha }$; where u is the wind speed in m/s

z is the height in m

z_r_ is the new reference height [we assume it to be 150m]

u_r_ is the wind speed at a reference height z_r_

α is the empirical coefficient that depends on atmospheric stability; for neutral stability, α = 0.143.

**Figure:**
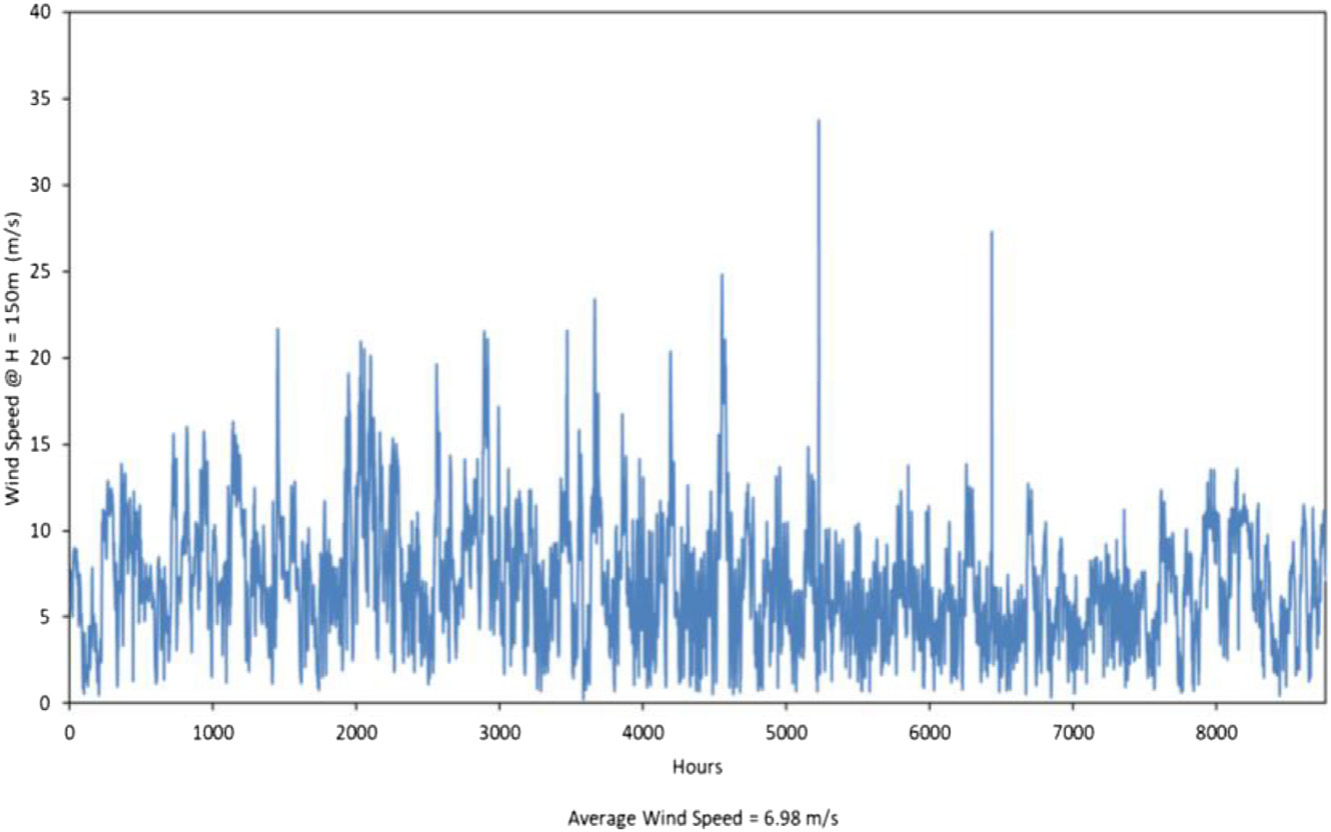

Annual hourly wind speed obtained from satellite data.

**Table:** SAM simulation parameters for PV, CSP, and Wind

Generation Technology	Parameters	Value
**PV**	Module Type	LG c-Si bifacial
	Efficiency (%)	20.33
	Albedo	0.3
	Bifaciality (%)	65
	Module height (m)	1
	Module rated power (W)	335
	Module dimensions (m)	1.674 ∗ 0.985
	Module array	18 ∗ 166
	Nameplate DC capacity (MW)	1
	Inverter power_AC_ (W)	40000
	Number of inverters	25
	Shading type	Standard non-linear
**CSP**	Collector type	Sky Fuel SkyTrough (with 80-mm OD receiver)
	Thermal to Electric Efficiency (%)	0.9
	Cycle thermal efficiency (%)	0.38
	HTF fluid	Therminol VP-1
	HTF pump efficiency (%)	85
	Total aperture area (m^2^)	443233
	Actual thermal output (MW_th_)	292.11
	Nameplate power output (MW_el_)	99.9
**Wind**	Wind turbine	GE 1.5sle
	Rotor diameter (m)	77
	Hub height (m)	80
	Turbine array	1 ∗ 1
	Nameplate capacity (MW)	1.5

Figure below shows the different demand profiles, irradiance and the generation from PV, CSP and Wind.

**Figure:**
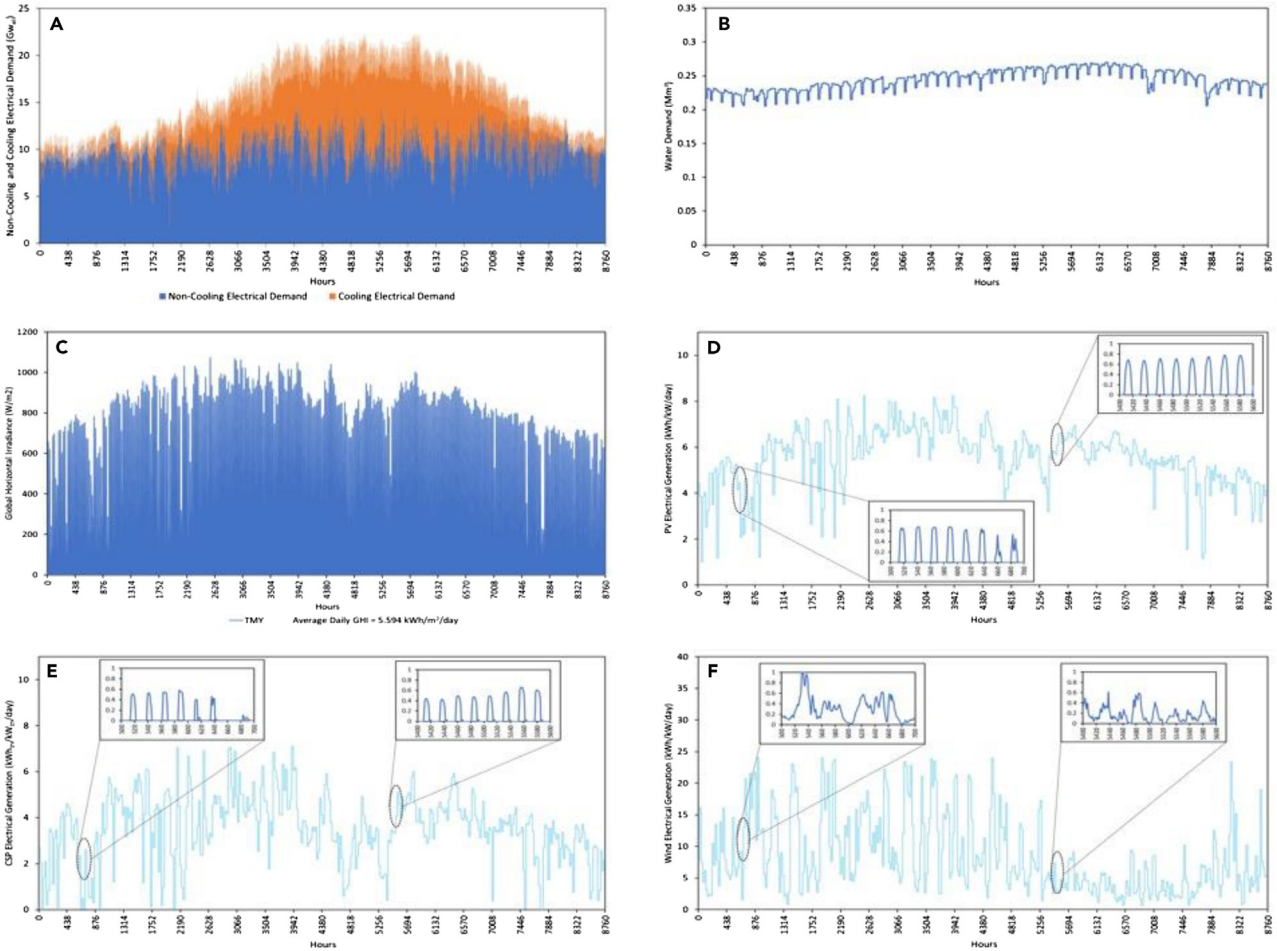

(a) Annual Hourly Non-Cooling Electric (Blue) and Cooling (Orange) Demand Profiles Synthesized for the UAE (2019 (see demand profiles creation section under STAR Methods)); (b) Annual Hourly Water Demand Profile Synthesized for the UAE (2019 (see demand profiles creation section under STAR Methods)); (c) Hourly GHI profile for TMY (see weather file creation section under STAR Methods); (d) Normalized daily sums of PV; € CSP; and (f) Wind Electricity Generation based on the TMY weather file. The magnified profiles show the hourly intensities for eight days in the summer and winter.

#### Different solar and wind project locations in the UAE

With the UAE rapidly advancing towards its clean targets of 2050, a few solar and wind sites that currently operational with a couple more in development. Figures below show the existing and planned solar and wind project locations in the UAE.

**Figure:**
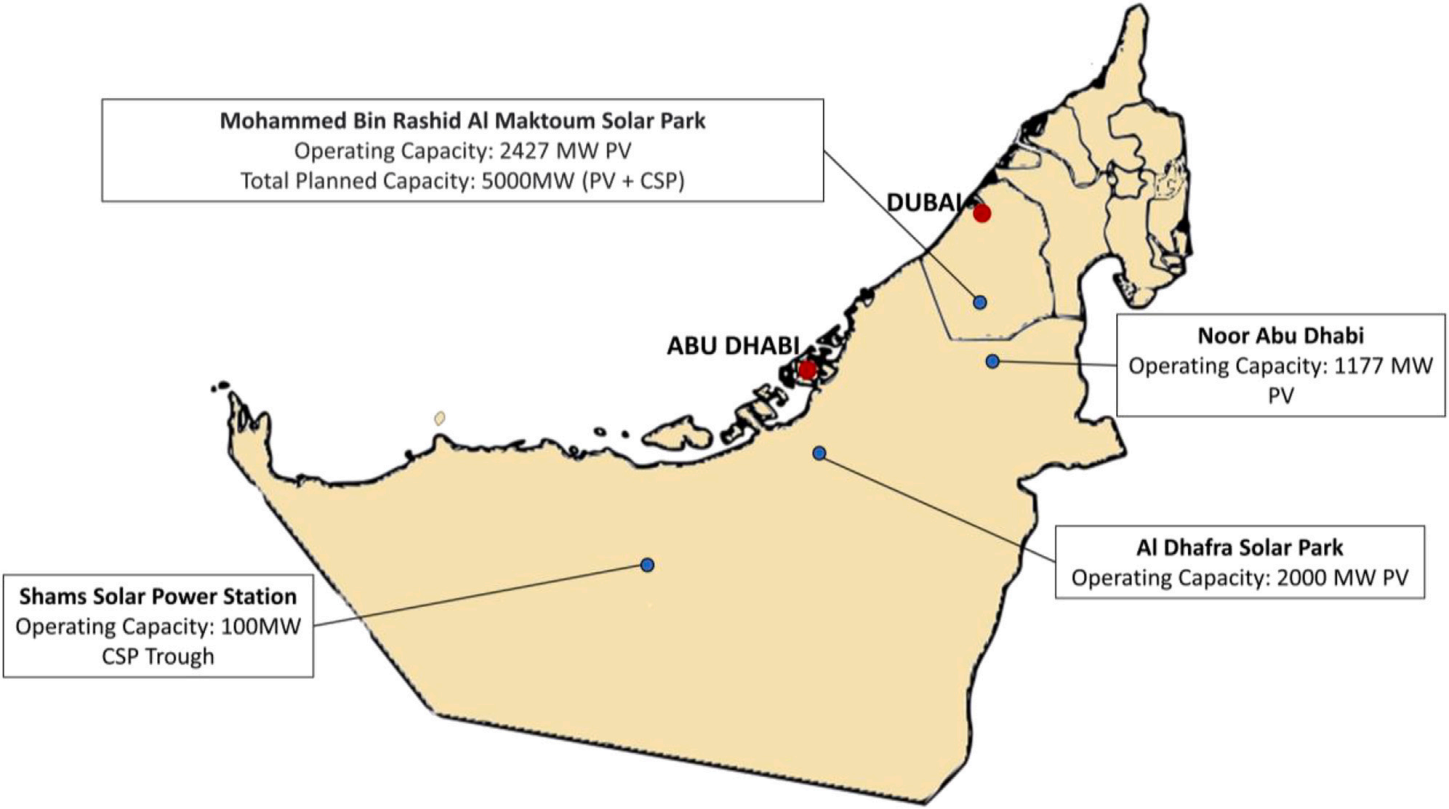

Locations of major solar energy projects in the UAE.

**Figure:**
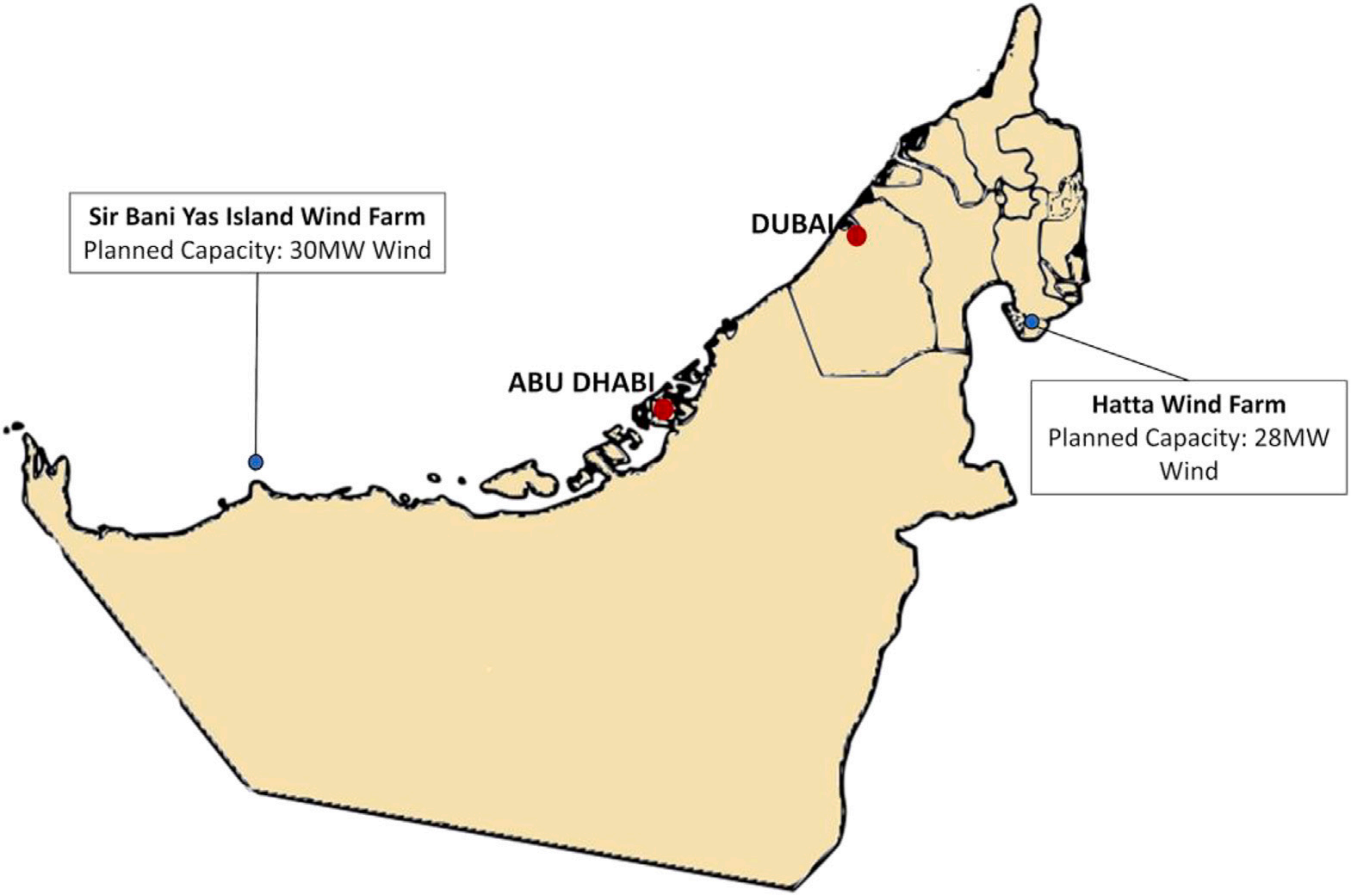

Locations of planned wind projects in the UAE^87^^,^^88^

#### Optimization model

To carry out this study we construct a linear optimization model of the energy system in Python, using a standard optimization package from Gurobi.^69^ We undertake a constrained linear optimization of the electricity system with the technology options, hourly demand, and RE supply profiles presented in the previous section for one year (see renewable energy profiles section under STAR Methods). The model offers a generalized version applied to the entire energy system of the approach used previously.^89^^,^^90^ The objective function of our optimization model is the minimization of the total annualized cost across the energy/water system (Equation 1). The function incorporates fixed & variable costs of all generation, storage, and flexibility technologies, with initial costs of technologies amortized based on the expected lifetime and a single discount rate.

(Equation 1) $$cost=\sum_{s=1}^{S}\left({FCSPD}_{s}\cdot {Cap}_{{discharge}_{s}}+{FCSPC}_{s}\cdot {Cap}_{{charge}_{s}}+{FCSC}_{s}\cdot {Cap}_{{storage}_{s}}\right)+\sum_{d=1}^{D}\left(\sum_{t=1}^{t}VC_{d}\cdot disp{out}_{d}\left(t\right)+{FC}_{d}\cdot disp{cap}_{d}\right)+\sum_{t=1}^{T}{PPA}_{nuclear}\cdot nuclear{out}_{c}\left(t\right)+\sum_{r=1}^{R}\sum_{t=1}^{T}{PPA}_{r}\cdot {renout}_{r}\left(t\right)$$

This equation is subject to a number of constraints, which represent the various operational requirements of the system and all of the technical or physical descriptions of each technology’s operation. Constraints are shown mathematically along with their brief descriptions in the table shown below. Some of the most important constraint functions are: C7 - controls the storage levels; C9 to C12 - ensures the balancing of demand and supply at any given hour for all four demand categories (non-cooling electric, cooling, water, and hydrogen); C13 - indicates the addition of Reserve Margin (RM).

Dispatchable power options ‘*d’* are CCGTgas (1), and GTgas (2) and Gas ST (3), clean energy power option ‘*c’* is Nuclear, and the variable renewable options ‘*r’* include PV (1), CSP (2), and Wind (3). The storage options ‘*s’* index represents Battery (1), Pumped Hydro (2), ACAES (3), Molten Salt (4), TES_cooling_ (5), Water _RO_ (6), Hydrogen (7), and Water _MSF_ (8). In order to properly represent storage, upstream processes are accounted for. For example, the charging capacity of TES_cooling_ storage includes all chiller capacity required to meet cooling demand; the charging capacity of water storage includes all desalination capacity, which necessitates defining a different water storage variable for each type of desalination technology used. The values in storage represent the corresponding actual physical form of storage (i.e., water, thermal energy, electricity, etc.). For this reason, the capacity/power values of the storage charging are also in the storage units. The costs of energy storage are energy specific.^91^ For representing discharge, we use the units of the final product. For example, water storage charge and discharge capacity have units of million cubic meters per hour (Mm^3^/h) – physically these would correspond to the desalination plant and distribution system capacity (i.e., pumps and pipes) respectively. A reserve margin of 25%^17^ applied to dispatchable capacities and storage levels is included in the optimization model (Constraints 13.1 and 13.2 in *Optimization Constraints* Table below) to ensure the robustness of the electricity supply in line with applicable practice.^92^

**Table:** Optimization constraints

Constraint No:	Constraint	Explanation
**1**	${supplyout}_{G}\left(t\right)\leq cap_{G}$	Output of any generator G cannot exceed generator capacity at any time
**2**	${storin}_{s}\left(t\right)={\eta c}_{s}*{{cap}_{charge}}_{s}$	Energy entering storage system S cannot exceed storage charging capacity
**3**	$curt_{r}\left(t\right)\leq {supplyout}_{r}\left(t\right)*{cap}_{r}\;\forall \;t$	Output of renewable source r can be curtailed up to total output
**4**	$storout_{s}\left(t\right)\leq {{cap}_{discharge}}_{s}/{\eta d}_{s}$	Energy delivered by storage system S cannot exceed storage discharging capacity
**5**	$\left(supplyout_{2}\left(t\right)-{curt}_{2}\left(t\right)\right)*{\eta c}_{4}={storin}_{4}\left(t\right)$	CSP-TES charges only from CSP solar field
**6**	$eleccooldem\left(t\right)*{\eta c}_{5}+storin_{5}\left(t\right)\leq {cap}_{{charge}_{5}}$	Sizes chillers to meet all direct cooling demand plus chilled water delivered to storage
**7**	$\sum_{s=1,2,3,7}\frac{storin_{s}\left(t\right)}{{\eta c}_{s}}\leq \sum_{r=1,3}{\theta_{r}*renout}_{r}\left(t\right)\;*r{encap}_{r}$	Electrical storage (battery, PHS, CAES, H_2_) charge only from renewable
**8.1**	$storlevel_{s}\left(t\right)=\left(1-selfdis_{s}\right)*storlevel_{s}\left(t-1\right)-storout_{s}\left(t\right)+{storin}_{s}\left(t\right)\;if\;t>0$ $storlevel_{s}\left(0\right)=\left(1-selfdis_{s}\right)*storlevel_{s}\left(8759\right)-storout_{s}\left(0\right)+storin_{s}\left(0\right)$	Storage balance between consecutive hours
**8.2**	$0\leq storlevel_{s}\left(t\right)\leq {cap}_{{storage}_{s}}$	Storage level cannot exceed storage capacity
**9**	$\sum_{d}{supplyout}_{d}\left(t\right)+{supplyout}_{c}\left(t\right)+\sum_{r=1,3}{\theta_{r}*supplyout}_{r}\left(t\right)+\sum_{s=1,2,3,4}{\eta d}_{s}*storout_{s}\left(t\right)+{\eta d}_{7}*\left({storout}_{7}\left(t\right)-hydrogendem\left(t\right)\right)==\frac{\left[elecdem\left(t\right)+eleccooldem\left(t\right)+\frac{storin_{5}\left(t\right)}{{\eta c}_{5}}\right]}{\left(1-transloss\right)}+\sum_{s=1,2,3,6,7}\frac{storin_{s}\left(t\right)}{{\eta c}_{s}}+\sum_{r=1,3}{curt}_{r}\left(t\right)$	Electrical demand satisfaction
**10**	$\left({\eta d}_{6}*{storout}_{6}\left(t\right)\right)+\left({\eta d}_{8}*{storout}_{8}\left(t\right)\right)==waterdem\left(t\right)/\left(1-waterloss\right)+\left({storin}_{7}\left(t\right)*\eta_{water\;to\;H2}\right)$	Water demand satisfaction $\eta_{water\;to\;H2}=0.00022817$ (1m^3^ water to 1kW_th_ of H_2_)
**10.1**	${storin}_{6}\geq 0.5*{{cap}_{charge}}_{6}$	RO cannot be operated below 50% capacity
**10.2**	$\frac{storin_{8}\left(t\right)}{{\eta c}_{8}}={{te}_{ratio}*dispout}_{1}\left(t\right)$	MSF is fed by waste heat from CCGT only. $te_{ratio}=0.6$ (Thermal heat to Electricity Ratio for MSF)
**11**	${\eta d}_{5}*{storout}_{5}\left(t\right)+eleccooldem\left(t\right)*{\eta c}_{5}=cooldem\left(t\right)$	Cooling demand satisfaction
**12**	$storout_{7}\left(t\right)\geq hydrogendem\left(t\right)\;\forall \;t$	H_2_ demand satisfaction
**13.1.1** **13.1.2** **13.2**	${storavail}_{s}\left(t\right)\leq {nd}_{s}*\left({storlevel}_{s}\left(t-1\right)\;if\;t>0\;else\;storlevel_{s}\left(8759\right)\right)$ $storlevel_{s}\left(init\right)={storlevel}_{s}\left(8759\right)\;\forall \;s$ ${storavail}_{s}\left(t\right)\leq \left({nd}_{s}*{storpd}_{s}\right)\;\forall \;s,t$ $\left(\left(\left(elecdem\left(t\right)+eleccooldem\left(t\right)+\frac{storin_{5}\left(t\right)}{{\eta c}_{5}}\right)/\left(1-transloss\right)\right)+\frac{storin_{6}\left(t\right)}{{\eta c}_{6}}\right)*\left(1+RM\right)-\left(\sum_{r=1,3}{\theta_{r}*renout}_{r}\left(t\right)*r{encap}_{r}\right)\leq dispcap_{d}+nuclearcap_{c}+\sum_{s=1,2,3,4,7}{storavial}_{s}\left(t\right)\;\forall \;t$	Available energy in a given storage at a given hour should be always the minimum of the storage level or the discharge capacity. Reserve Margin (RM) is added to the storage in regard to electricity

#### LCOE and amortized cost assessment for different generators and storages

In order to evaluate the objective function, we specify each cost component included in Equation 1. For technology costs, we rely on available local project costs and international reporting for technologies not currently deployed in the UAE. We note that the costs of renewable energy technologies, which over the last decade were characterized by aggressive reductions, have plateaued recently; this can be attributed to inflationary pressures across the global economy as well as specific supply chain strains.^47^ As of Q1 2024, these pressures appear to be easing and module prices have resumed a downward trajectory.^93^^,^^94^ Additional price reductions may occur in the future as technologies further mature and scale up,^48^ but recent history offers a note of caution against overoptimistic projections of future costs. Of several price evolution scenarios considered, we have used the most conservative (highest prices for renewable/storage technology) for our analysis. For nuclear power, we consider LCOE of 5.6¢/kWh (assumptions stated in *Cost Assessment – Renewable and Nuclear Generators* Table) in both current and future scenarios, since conventional nuclear has not exhibited learning behavior over recent decades and is heavily dependent on the local regulatory and financial environment. Several energy sources are treated as independent power producers (IPPs) whose cost is defined only by a power purchase agreement (PPA) in $/kWh. This largely matches the structure under which these plants are constructed and operated in the UAE for RE power with existing power purchase agreements (PPAs) history, which is PV. For CSP, we use a calculated cost of 0.014$/kWh_th_ (see *Cost Assessment – Renewable and Nuclear Generators* Table) and then put the rest of the cost into storage (see *CAPEX and Cost Assessment – Thermal (CSP Molten Salts)* Tables). For wind, which has no registered project in the country, we combine the generation profile with the financial system data (cost of capital, lifetime, construction and maintenance costs, interest rate adjusted to the region) in estimating the Levelized Cost of Electricity (LCOE) (see *Cost Assessment – Renewable and Nuclear Generators* Table).

GT_gas_, CCGT_gas_ and Gas ST capacity, as well as storage technologies, are fully modeled based on CAPEX, fixed OPEX, and variable costs. We treat the CAPEX as three separate components: charging, storage, and discharging CAPEX in $/kW_charge_, $/kWh_capacity_, and $/kW_discharge_, respectively. Each of these in general refers to a different set of hardware. For example, when considering hydrogen storage, the charging capacity is the electrolyzer, the storage capacity is the tank or cavern, and the discharge capacity is the fuel cell or engine that converts hydrogen back into electricity. The description of each storage component along with their efficiencies (these include unit conversion factors if energy is converted between forms with different units) is presented in the methodology (see storages section under STAR Methods). Once the different generator and storage capacities are obtained along with the total system cost, we remove the amortized costs corresponding to the charging, storage and discharging components of TES_cooling_ (Ice/chilled water), hydrogen that is re-electrified, and water (RO and MSF) to find out the actual cost of electricity generation and consumption as shown in results.

$${LCOE}_{renewables\;\&\;nuclear}=\frac{C_{0}+\sum_{i=1}^{L}\frac{C_{i}}{{\left(1+r\right)}^{i}}}{\sum_{i=1}^{L}\frac{E_{i}}{{\left(1+r\right)}^{i}}}$$

$${AFC}_{gas\;generators\;and\;storage\;components}=C_{o}*\;\left(\frac{{r\left(1+r\right)}^{L}}{{\left(1+r\right)}^{L}-1}\right)+\left(C_{o}*\;C_{i}\right)$$

$${VC}_{gas\;generators}=\frac{gas\;price}{293.01*\eta }$$

LCOE– Levelized cost of electricity ($/kWh)

AFC– Amortized fixed cost

VC– Variable cost

$C_{0}$ – Initial Investment/CAPEX ($)

L– Lifetime (years)

$C_{i}$ – Annual fixed cost/OPEX (%)

r – Discount rate (%)

$E_{i}$ – Annual energy output (kWh)

η– Plant efficiency (%)

#### Generators

**Table:** Cost assessment – Renewable and nuclear generators

Metrics	Generators							
	Renewables						Nuclear	
	PV		CSP		Wind			
**CAPEX ($)**	–	–	403.5	DEWA^95^	1250	Energy^96^	4357	ENEC^97^
**Fixed OPEX (%)**	–	–	4	IRENA^98^	2	Apostoleris et al.^99^	2	Apostoleris et al.^99^
**Discount rate (%)**	–	–	4	Apostoleris et al.^99^	4	Apostoleris et al.^99^	8^a^	–
**Capacity factor**	–	–	0.3	–	0.35	–	0.9	–
**Lifetime**	–	–	35	DEWA^100^	20	Energy^96^	60	Technology^101^
***2021 Calculated LCOE/PPA (if applicable)***	*1.6 ¢/kWh*	DEWA^102^	*1.4 ¢/kWh*_*th*_	*-*	*3.8 ¢/kWh*	*-*	*5.6 ¢/kWh*	*-*
***2050 Calculated LCOE/PPA (if applicable)***	*1 ¢/kWh*	IRENA^103^	*0.8*^b^*¢/kWh*_*th*_	*-*	*2 ¢/kWh*	Way et al.^104^	*5.6 ¢/kWh*^c^	*-*

a First of its kind project in Middle East, hence we estimate a higher DR.

b Based on IEA CSP capacity projections^105^ and IRENA CSP CAPEX learning curves.^8^ (see IEA CSP Capacity projections and IRENA CSP CAPEX Learning Curve Figure).

c Based on,^104^ learning curve does not exhibit reduction in nuclear costs since they are heavily dependent on a country’s policy, and since this is the first of its kind project in the Middle East, the cost is estimated to be the same.

**Table:** Cost assessment – Gas generators

Metrics	Generators^a^					
	CCGTgas		GTgas		Gas ST^b^	
**2021 and 2050 CAPEX ($)**	771	DEWA^106^	360	DEWA^107^	1349^c^	DEWA^108^
**Lifetime (years)**	30	d	30	d	25	DEWA^108^
**Fixed OPEX (%)**	2	Apostoleris et al^99^	2	Apostoleris et al^99^	2	Apostoleris et al.^99^
**Discount rate (%)**	5	Apostoleris et al.^99^	5	Apostoleris et al^99^	5	Apostoleris et al.^99^
**Efficiency (%)**	48	Repowering Jebel Ali D Station^109^	37	De Sa and Al Zubaidy^110^	40	
**Gas Price**	4	EIA^111^	4	EIA^111^	4	EIA^111^
***Calculated AFC ($/kW)***	*65.57*	*-*	*30.62*	*-*	*122.39*	*-*
***Calculated VC ($/kWh)***	*0.028*	*-*	*0.037*	*-*	*0.034*	*-*

a Based on past 26 years historical average,^111^ gas price of 4 $/MMBtu is used throughout the study.

b This is a clean generator with carbon capture system (CCS).

c This includes the cost of CCS system (642 $/kW).^112^

d Based on the time between refurbishing the existing power plants.

#### Storages

**Table:** Storage technologies with component breakdown (charging, storage and discharging)

Storage Technology	Storage Type	Power Component (Charge)	Energy Component (Storage)	Power Component (Discharge)
Lithium Ion Battery	Electromechanical	Inverter	Battery Cells	Rectifier
Pumped Hydro Storage (PHS)	Mechanical	Pump/Reversible Turbine	Reservoir	Turbine
Adiabatic Compressed Air Energy Storage (ACAES)	Mechanical	Compressor	Caverns	Turbine
Thermal (CSP Molten Salts)	Thermal	Thermal Oil Loop	Tanks	Power Block (Turbine)
Ice / Chilled Water Storage	Thermal	Chiller	Tanks	Heat Exchanger
Water Desalination (RO)	Water	Reverse Osmosis (RO) system	Reservoirs	Pump
Water Desalination (MSF)	Water	Multistage Flash (MSF) system	Reservoirs	Pump
Hydrogen (H_2_)	Chemical	Electrolyzer	Caverns	Fuel Cell

#### Lithium ion battery

**Table:** Cost assessment – Lithium ion battery storage

Metrics	Lithium-Ion Battery Storage											
	2021						2050					
	Charging		Storage		Discharging		Charging		Storage		Discharging	
**CAPEX ($)**	60^a^	NREL^113^	220	NREL^114^	60^b^	NREL^113^	30^c^	–	62	NREL^114^	30^c^	–
**Efficiency (%)**	93	Kebede et al.^115^	N/A	–	93	Kebede et al.^115^	93	–	N/A	–	93	–
***Calculated AFC***	*8.4*	*-*	*30.9*	*-*	*8.4*	*-*	*4.2*	*-*	*8.7*	*-*	*4.2*	*-*

Lifetime = 11 years^89^; OPEX = 2%; Discount Rate = 5%.

a Based on inverter price from.^113^

b Based on electrical BOS from.^113^

c Based on 2021 charging/discharging cost^113^ and learning curve from.^116^

#### Pumped Hydro Storage (PHS)

**Table:** Cost assessment – pumped hydro storage

Metrics	Pumped Hydro Storage (PHS)											
	2021						2050					
	Charging		Storage		Discharging		Charging		Storage		Discharging	
**CAPEX ($)**	131^a^	–	42	Mongird et al.^117^	1177^b^	–	131^c^	–	42^c^	–	1177^c^	–
**Efficiency (%)**	90	Kebede et al.^115^	N/A	–	90	Kebede et al.^115^	90	–	N/A	–	90	–
***Calculated AFC***	*9.3*	*-*	*3*	*-*	*83.6*	*-*	*9.3*	*-*	*3*	*-*	*83.6*	*-*

Lifetime = 80 years^118^; OPEX = 2%; Discount Rate = 5%.

Charging cost = (power cost/charging capacity [0.25GW]/(charge cost ratio).

Discharging cost = (power cost/charging capacity [0.25GW]/(discharge cost ratio).

a and b Based on^117^ we obtain power cost [327 m$] by removing storage CAPEX [42$/kWh ∗ 1.5GWh storage capacity] from total PHS CAPEX [390 m$]^118^; and assume the discharge to charge component cost as 10:1.

c As per^104^ we see that there is no significant decrease in the learning rate, hence the costs in 2050 are kept to be the same as 2021.

Based on ANU’s potential PHS sites mapping tool,^119^ we set the upper limit on PHS storage to be 10GWh for our study.

#### Adiabatic Compressed Air Energy System (ACAES)

**Table:** CAPEX assessment – Adiabatic compressed air energy system

Metrics	Adiabatic Compressed Air Energy Storage (ACAES) CAPEX Analysis			
	Charging	Storage	Discharging	Reference
**Cost (m€)**	22.6	79	25.9	Huang et al.^120^
**Cost**^a^**(m$)**	25.54	89.27	29.27	–
**Size (MW)**	103	854	140	Huang et al.^120^
***CAPEX***^b^***($)***	*248*	*105*	*209*	*-*

a 1€ = 1.17$ (as per 2017; this is the year when the study in the published paper was conducted^120^).

b CAPEX = (cost(m$)∗(1000/size)).

**Table:** Cost assessment – Adiabatic compressed air energy system

Metrics	Adiabatic Compressed Air Energy Storage (ACAES)											
	2021						2050					
	Charging		Storage		Discharging		Charging		Storage		Discharging	
**CAPEX ($)**	248	Huang et al.^120^	105	Huang et al.^120^	209	Huang et al.^120^	248^a^	–	105^a^	–	209^a^	–
**Efficiency (%)**	74	Kebede et al.^115^	N/A	–	88	Kebede et al.^115^	74	–	N/A	–	88	–
***Calculated AFC***	*21.1*	*-*	*8.9*	*-*	*17.8*	*-*	*21.1*	*-*	*8.9*	*-*	*17.8*	*-*

Lifetime = 30 years^121^; OPEX = 2%; Discount Rate = 5%.

a As per,^121^ we see that there is no significant decrease in the cost, hence the costs in 2050 are kept to be the same as 2021.

#### Thermal (CSP Molten Salts)

**Table:** CAPEX assessment – Thermal (CSP Molten Salts)

Metrics	CSP CAPEX Analysis	Reference
Trough Model Cost ($/kW)	4449	IRENA^8^
Solar Field Cost ($/kW)	1345	IRENA^8^
HTF (Charging) Cost ($/kW)	470	IRENA^8^
TES (Storage) Cost ($/kW)	660	IRENA^8^
Power Block (Discharging) Cost ($/kW)	834	IRENA^8^
Duration of TES (Storage) (hours)	*6*	IRENA^8^
TES (Storage) Cost ($/kWh)	*110*	*-*
Solar Field Cost ($/kW_th_)^a^	*403.5*	*-*

a (Solar Field Cost ($/kW) ∗ Discharge Efficiency).

**Table:** Cost assessment – Thermal (CSP Molten Salts)

Metrics	Molten Salt Storage (TES)											
	2021						2050					
	Charging		Storage		Discharging		Charging		Storage		Discharging	
**CAPEX ($)**	141^a^,∗	–	33^b^,∗	–	834	–	82^c^,∗	–	19^c^,∗	–	486^c^	–
**Efficiency (%)**	98	Kebede et al.^115^	N/A	–	30	Kebede et al.^115^	98	–	N/A	–	30	–
***Calculated AFC***	*11.4*	*-*	*2.7*	*-*	*67.6*	*-*	*6.6*	*-*	*1.5*	*-*	*39.4*	*-*

Lifetime = 35 years^95^; OPEX = 2%; Discount Rate = 5%.

a (HTF (Charging) Cost ($/kW) ∗ Discharge Efficiency).

b (TES (Storage) Cost ($/kWh) ∗ Discharge Efficiency).

c Based on IEA CSP capacity projections^105^ and IRENA CSP CAPEX learning curves^8^ (see *IEA CSP Capacity projections and IRENA CSP CAPEX Learning Curve* Figure).

∗ Thermal Units.

2050 CSP – TES component costs are projected using IEA CSP capacity projections^105^ and IRENA CSP CAPEX learning curves^8^ as shown in Figure below.

**Figure:**
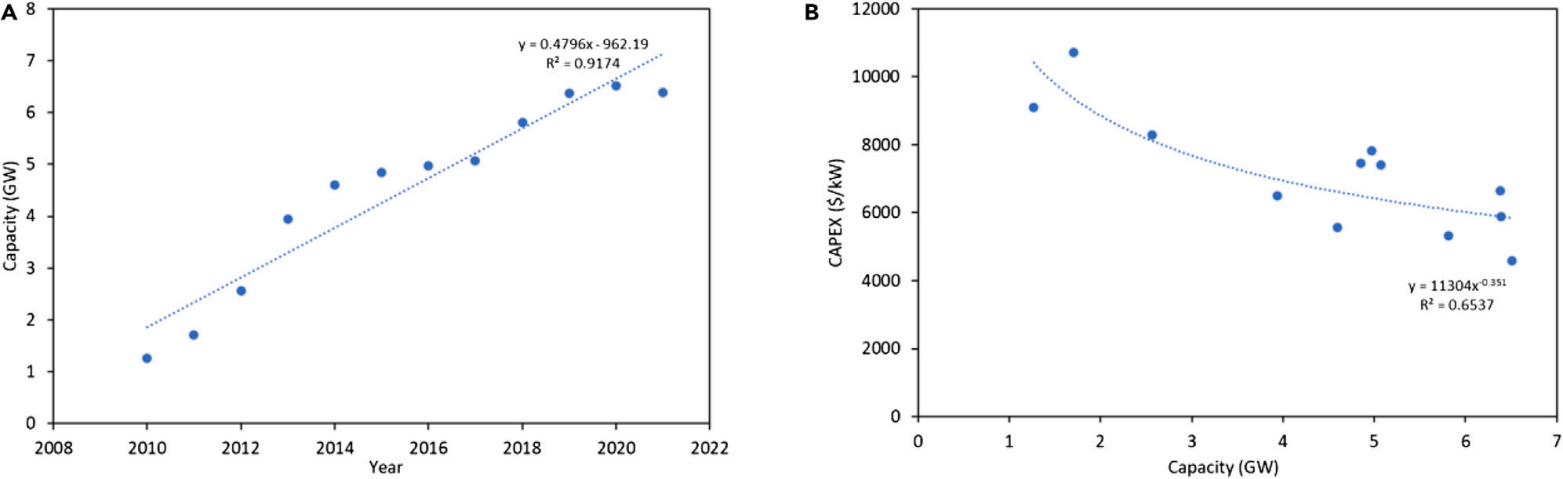

a) IEA CSP capacity projections and b) IRENA CSP CAPEX learning curves.

#### Ice/Chilled water storage

**Table:** Cost assessment – Ice / chilled water storage

Metrics	Ice Storage (TES_cooling_)											
	2021						2050					
	Charging		Storage		Discharging		Charging		Storage		Discharging	
**CAPEX ($)**	293^a^	–	15^b^	Erik Nielsen et al.^122^	0^c^	–	293^d^	–	15^d^	–	0^d^	–
**Efficiency (%)**	280	Yu et al.^73^	N/A	–	90	Kebede et al.^115^	280	–	N/A	–	90	–
***Calculated AFC***	*24.9*	*-*	*1.3*	*-*	*0*	*-*	*24.9*	*-*	*1.3*	*-*	*0*	*-*

Lifetime = 30 years^123^; OPEX = 2%; Discount Rate = 5%.

a Based on,^76^ and conversion from kW_th_ to RT [3.52].

b See Figure 4.4 from^122^.

c Cost of discharge equipment of TES_cooling_ storage is already included in the storage price.

d Due to limited data availability owing to the slow rate of adoption of the technology, the costs for 2050 are kept being the same as 2021.

#### Water desalination (RO and MSF)

**Table:** Cost assessment – Water desalination (RO and MSF)

Metrics	Water Desalination (RO and MSF)															
	2021								2050							
	RO Charging		MSF Charging		Storage		Discharging		RO Charging		MSF Charging		Storage		Discharging	
**CAPEX ($)**	26759^a^	–	40481^b^	–	174^c^	–	0^d^	–	13379.5	Caldera and Breyer^124^	40481^e^	–	174	–	0	–
**Efficiency (%)**	25^f^	–	1.25^g^	–	N/A	–	99^c^	–	25	–	1.25	–	N/A	–	99	–
***Calculated AFC***	*2275.9*	*-*	*3443*	*-*	*14.8*	*-*	*0*	*-*	*1137.9*	*-*	*3443*	*-*	*14.8*	*-*	*0*	*-*

Lifetime = 30 years; OPEX = 2%; Discount Rate = 5%.

a Desalination Capacity = 180 MIGD^78^; MIG to Mm^3^ = 220; Project CAPEX = 912 m$^78^.

b Desalination Capacity = 140 MIGD^106^; MIG to Mm^3^ = 220; Project CAPEX = 1073 m$^106^.

c Storage Capacity = 60 MIGD^125^; MIG to Mm^3^ = 220; Project CAPEX = 48 m$.^125^

d Water flows through pipes mostly, hence no cost for discharge and very high efficiency.

e The cost ($/(m^3^/h)) calculated is similar to the 15 year old low end plant cost of the desalination facility from Texas.^126^ This shows that there is not much disparity between the costs over the 15 years, hence they are not expected to change much in the future.

f 4 KWh is required by the RO system to produce 1m^3^ of water. ∴ Charging efficiency of RO = (1/4).

g 60 KWh is required by the MSF system to produce 1m^3^ of water and the conversion factor of heat in MSF plants for desalination is estimated to be 60%. ∴ Charging efficiency of MSF = ((1∗0.6)/60).

#### Hydrogen

**Table:** Cost assessment – Hydrogen

Metrics	Hydrogen (H_2_)											
	2021						2050					
	Charging		Storage		Discharging		Charging		Storage		Discharging	
**CAPEX ($)**	1000	Gandhi et al.^60^	5.5	Guerra et al.^127^	1500	Whiston et al.^128^	300	Gandhi et al.^60^	1.5	Guerra et al.^127^	800	Whiston et al.^128^
**Efficiency (%)**	70	Gandhi et al.^60^	N/A	–	60	Osman et al.^89^	70	–	N/A	–	60	–
***Calculated AFC***	*116.3*	*-*	*0.4*	*-*	*174.5*	*-*	*34.9*	*-*	*0.1*	*-*	*93.1*	*-*

Lifetime = 15 years^129^; OPEX = 2%; Discount Rate = 5%.

#### A note on the equivalence of PPA and CAPEX representations for variable renewable generators

We use a variable cost (PPA price per kWh) to represent the cost of PV and wind, representing the actual cost structure of many utilities that procure renewable energy from independent power producers, rather than building and owning wind and solar farms themselves. We show here that these two representations are mathematically equivalent. The PPA price is equal to the LCOE of a renewable facility with a given capacity cost CAPEX and fixed operating cost OPEX_annual_, calculated over the total lifetime generation of the plant, or:

$$PPA=\frac{CAPEX+\sum_{i=1}^{L}\frac{{OPEX}_{annual}}{{\left(1+r\right)}^{i}}}{\sum_{i=1}^{L}\frac{{Gen}_{annual}}{{\left(1+r\right)}^{i}}}$$

where *r* is the discount rate or IRR target, *L* is the lifetime of the plant and *Gen*_*annual*_ is the total energy generated by the plant in one year, which is determined by the pre-set solar profile. Evaluating the geometric series of x = 1/(1 + r), this becomes:

$$PPA=\frac{CAPEX+{OPEX}_{annual}\left(\frac{x\left(1-x^{L}\right)}{1-x}\right)}{{Gen}_{annual}\left(\frac{x\left(1-x^{L}\right)}{1-x}\right)}=\frac{CAPEX\left(\frac{1-x}{\left(x-x^{L+1}\right)}\right)+{OPEX}_{annual}}{{Gen}_{annual}}$$

The inverted geometric sum can be rearranged to:

$$\left(\frac{1-x}{x-x^{L+1}}\right)=\left(\frac{1-\left(1/\left(1+r\right)\right)}{\left(1/\left(1+r\right)-{\left(1/\left(1+r\right)\right)}^{L+1}\right)}\right)=\left(\frac{r{\left(1+r\right)}^{L}}{{\left(1+r\right)}^{L}-1}\right)$$

Which is the amortization factor applied to convert total capex into an annualized value for conventional generators and storage in our model. The annual cost associated with a renewable generator is the total generation multiplied by the PPA price – curtailed energy is still paid for and curtailment does not reduce annual cost. The annual cost for the renewable generator is therefore:

$$annual\;cost=PPA\times {Gen}_{annual}=CAPEX\left(\frac{r{\left(1+r\right)}^{L}}{{\left(1+r\right)}^{L}-1}\right)+{OPEX}_{annual}$$

which is identical to the annual fixed cost for any other component of the model. Hence the fixed costs are embedded in the PPA price and are not impacted by any operational decisions the model may take.
