# Supplementary material for: Realizing renewable resilience: Lessons from the Middle East for the global energy transition
Source: iScience. 2024 May 21;27(7):110058. doi: 10.1016/j.isci.2024.110058 (PMC11260868; doi:10.1016/j.isci.2024.110058)
Supplement: Document S1. Figure S1 and Table S1 [file mmc1.pdf]

iScience, Volume *27*

Supplemental information

Realizing renewable resilience: Lessons from the Middle East for the global energy transition

Ansari Aadil Shahzad, Abdullahi Bamigbade, Krishiv Gandhi, Juan David Barbosa, Andrei Sleptchenko, Harry Nicholas Apostoleris, and Sgouris Sgouridis

Supplemental Information

Realizing Renewable Resilience: Lessons from the Middle East for the Global Energy Transition

Ansari Aadil Shahzad[1], Abdullahi Bamigbade[2], Krishiv Gandhi[1], Juan David Barbosa [1], Andrei Sleptchenko[3], Harry Nicholas Apostoleris[1]*, Sgouris Sgouridis[1]

[1]*Dubai Electricity & Water Authority Research & Development Center, Dubai, UAE*

[2]*Tandon School of Engineering, New York University, New York, USA*

[3]*Khalifa University of Science & Technology, Abu Dhabi, UAE*

*harry.nicholas@dewa.gov.ae (Lead Contact)

S1. Supporting Results

S1.1 Worst years stress test against TMY for Scenarios 1 and 2 (shown in Main Text)

Table S1.1 *Worst years stress test against TMY for Scenarios 1 and 2 (shown in Main Text), Related to* ***Table 1 Optimized system configuration results across notable scenarios.*** *Sub tables displaying: a) generation capacities, b) storage technologies with charging, storage, and discharging capacities, and c) financial and d) CO2 metrics.*

.

**Scenarios**	**1 - 2025ext**				**2 - 2025opt**			
**Year**	**Design**	**Test**			**Design**	**Test**		
	**TMY**	**2006**	**2011**	**2019**	**TMY**	**2006**	**2011**	**2019**
**a) Financial and emissions metrics**								
**Total System Cost (billion $)**	10.897	10.838	10.847	10.844	10.444	10.438	10.442	10.437
**Curtailment (GWh)**	4145.4	2084	2563.4	258.3	4547.6	3872.6	3833.8	3882.3
**Cost of Electricity Generation ($/MWh)**	48.6	48.8	48.7	48.8	45.6	45.8	45.7	45.7
**Cost of Electricity Consumption ($/MWh)**	52.4	52.1	52.1	52.1	49.3	49.3	49.2	49.3
**CO_2 emissions (tons/GWh generated)**	242	241	242	241	157	161	161	160
**CO_2 emissions (tons/GWh consumed)**	261	257	258	258	169	173	173	173

S1.2 Stress test against worst year for Scenarios 4 and 5 (shown in Main Text)

Table S1.2 *Stress test against worst year for Scenarios 4 and 5 (shown in Main Text), Related to* ***Table 1 Optimized system configuration results across notable scenarios.*** *Sub tables displaying: a) generation capacities, b) storage technologies with charging, storage, and discharging capacities, and c) financial and d) CO2 metrics*

Scenarios	4 – 2050E0str			3 – 2050E0opt *	5 – 2050MIXstr		
Year	Design	Test		Design	Design		Test
	2006	2011	2019	TMY	2006	2011	2019
**a) Financial and emissions metrics**							
**Total System Cost (billion $)**	9.765	9.762	9.771	9.490	9.321	9.148	9.252
**Curtailment (GWh)**	65845.1	68742.1	69007.7	74822.9	61906.6	61024.1	61869.1
**Cost of Electricity Generation ($/MWh)**	32.4	32.5	32.4	30.6	31.9	31.3	31.6
**Cost of Electricity Consumption ($/MWh)**	49.1	49.6	49.7	48.1	47.5	46.4	46.9
**CO_2 emissions (tons/GWh generated)**	0	0	0	0	1	1	1
**CO_2 emissions (tons/GWh consumed)**	0	0	0	0	2	1	1

* This run fails the stress test against the worst years; hence we optimize for the worst year and stress test it against the other two worst years.

S1.3 Optimized results for Scenarios 2.1, 4.1, 4.2, 4.3, 4.4 and 4.5 (shown in Main Text).

Table S1.3 *Optimized results for Scenarios 2.1, 4.1, 4.2, 4.3, 4.4 and 4.5 (shown in Main Text), Related to Results and Discussion.*

Scenarios		2.1	4.1	4.2	4.3	4.4	4.5
**Name**		**2025opt.1**	**2050E0str.1**	**2050E0str.2**	**2050E0str.3**	**2050E0str.4**	**2050E0str.5**
**Description**		**Fixed $TES_{cooling}$ capacities from Scenario 2**	**Annual Green H_2 demand in Scenario 4**	**Battery storage costs to 220 $/kWh keeping other costs same as Scenario 4**	**Battery storage costs to 140 $/kWh keeping other costs same as Scenario 4**	**2021 and 2050 average H_2 storage components (charging, storage and discharging) costs keeping other costs same as Scenario 4**	**Cut 2050 H_2 storage components (charging, storage and discharging) costs by half keeping other costs same as Scenario 4**
**a) Generator Capacities**							
**Renewables**	**PV (GW)**	23.98	149.3	99.2	98.4	115.6	88.3
	**CSP (GW)**	0.7	0.7	0.7	0.7	0.7	0.7
	**Wind (GW)**	0	6	6	6	6	6
**Gas**	**CCGTgas (GW)**	25.8	0	0	0	0	0
	**GTgas (GW)**	4.62	0	0	0	0	0
	**Gas ST (GW)**	3.27	0	0	0	0	0
**Nuclear**	**Nuclear (GW)**	5.6	5.6	5.6	5.6	5.6	5.6
**b) Storage Capacities**							

Lithium Ion Battery	Charge (GW)	0	36.19	0	1.71	42.76	27
	Storage (GWh)	0	216.2	0	6.79	250.66	171.39
	Discharge (GW)	0	17.62	0	5.37	26.31	18.92
Pumped Hydro Storage (PHS)	Charge (GW)	0.3	0.56	2.22	2.22	0.55	0.46
	Storage (GWh)	1.62	7.25	10	10	9.75	3.75
	Discharge (GW)	0.25	0.25	0.67	0.67	0.25	0.25
Adiabatic Compressed Air Energy System (ACAES)	Charge (GW)	0	0	17.6	16.1	0	0
	Storage (GWh)	0	0	97.35	89.73	0	0
	Discharge (GW)	0	0	13.81	8.16	0	0
Thermal CSP (Molten Salts)	Charge (GW_{th})	5.25	5.25	5.25	5.25	5.25	5.25
	Storage (GWh_{th})	31.5	31.5	31.5	31.5	31.5	31.5
	Discharge (GW)	0.7	0.7	1.09	1.09	0.76	0.87
Chilled Water / Ice	Charge (GW)	45.8	43.22	75.33	75.42	41.32	41.44
	Storage (GWh_{th})	16.9	166.88	399.32	399.87	345.73	88.83
	Discharge (GW_{th})	7.1	17.22	42.99	42.17	26.09	15.81
Water Desalination	Charge RO (Mm^3h)	0.187	0.261	0.261	0.261	0.26	0.262
	Charge MSF (Mm^3h)	0.261	0	0	0	0	0
	Storage (Mm^3)	0.635	1.934	0.419	0.566	4.61	0.246
	Discharge (Mm^3h)	0.308	0.272	0.267	0.267	0.266	0.268
Hydrogen (H_2)	Charge (GW)	0	37.85	6.73	6.834	0.021	11.964
	Storage (GWh_{th})	0	3693.3	1407.8	1405.3	66.24	5522.4
	Discharge (GW)	0	13.38	8.21	8.18	0.513	12.001

c) Financial and emissions metrics						
Total System Cost (billion $)	10.539	12.661	10.471	10.423	9.683	8.829
Curtailment (GWh)	4663.2	52197.3	60218.7	59176.9	105376.5	32139.7
Cost of Electricity Generation ($/MWh)	46.2	31.2	29.4	29.4	26.9	31.9
Cost of Electricity Consumption ($/MWh)	49.9	39.9	46.3	46	47	44.6
CO_2 emissions (tons/GWh generated)	173	0	0	0	0	0
CO_2 emissions (tons/GWh consumed)	187	0	0	0	0	0

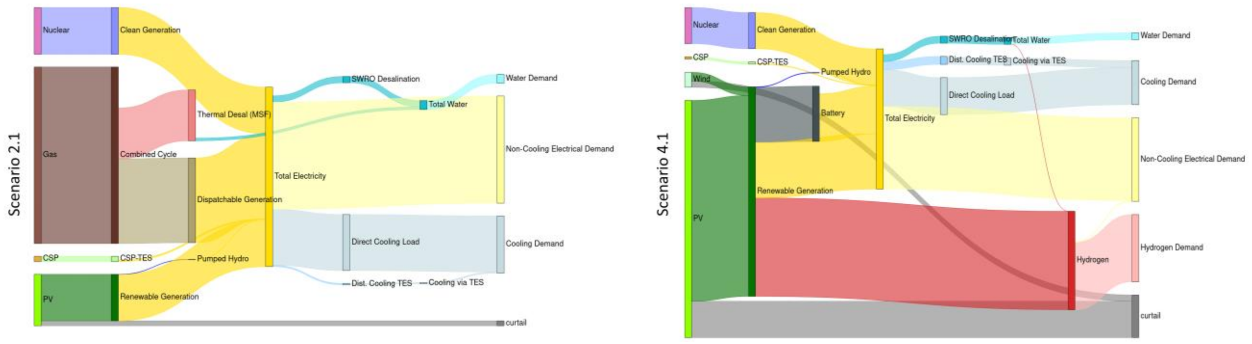

Figure S1.3.1 *Annual energy flows from various energy sources to satisfy respective demands directly or via storages across scenarios 2.1 and 4.1 outlined in Table S1.3, Related to Results and Discussion.*

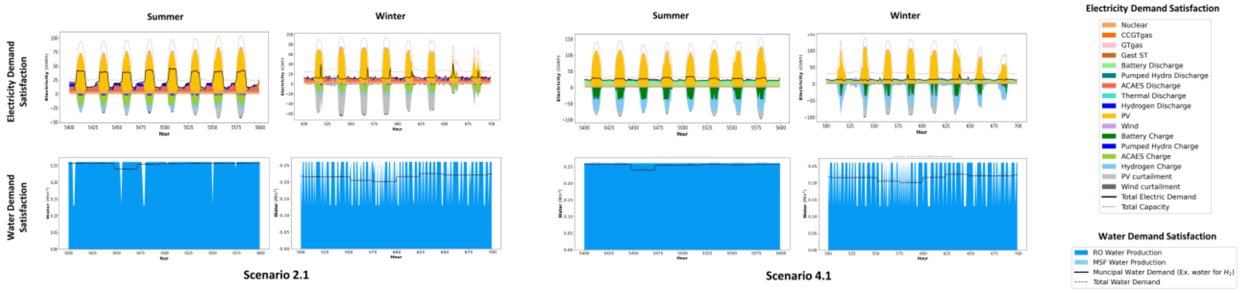

Figure S1.3.2 *Electricity and Water demand satisfaction figures in summer and winter across scenarios 2.1 and 4.1 outlined in Table S1.3, Related to Results and Discussion.*

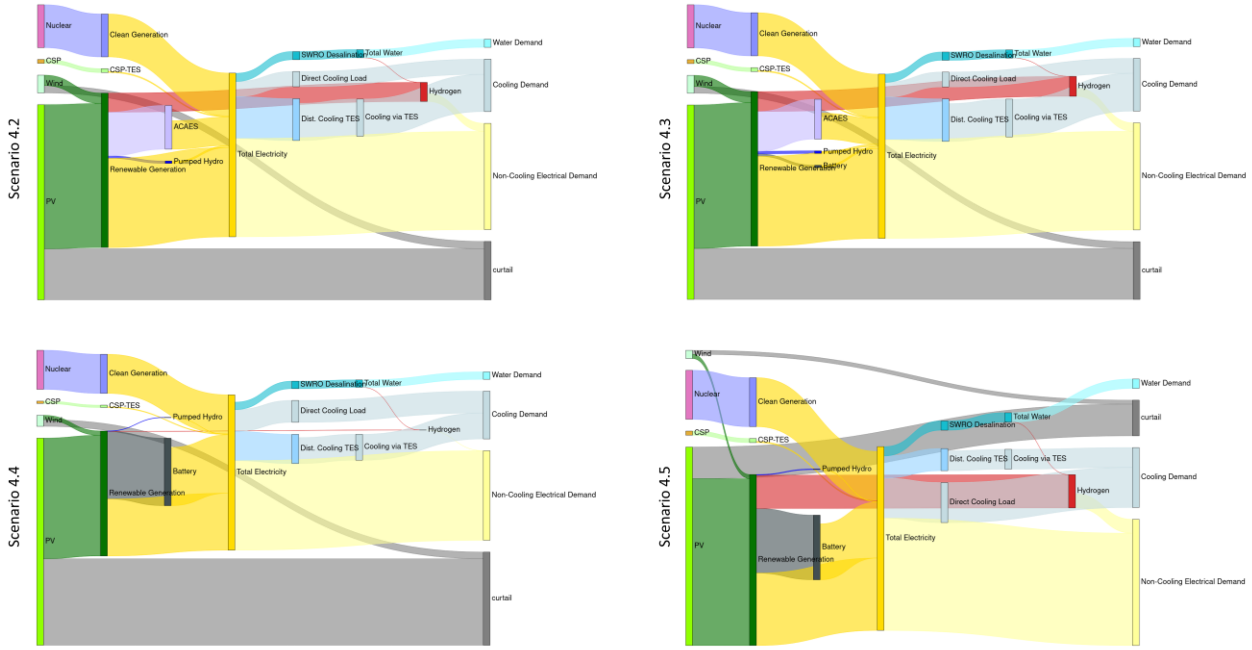

Figure S1.3.3 *Annual energy flows from various energy sources to satisfy respective demands directly or via storages across scenarios 4.2, 4.3, 4.4 and 4.5 outlined in Table S1.3, Related to Results and Discussion.*

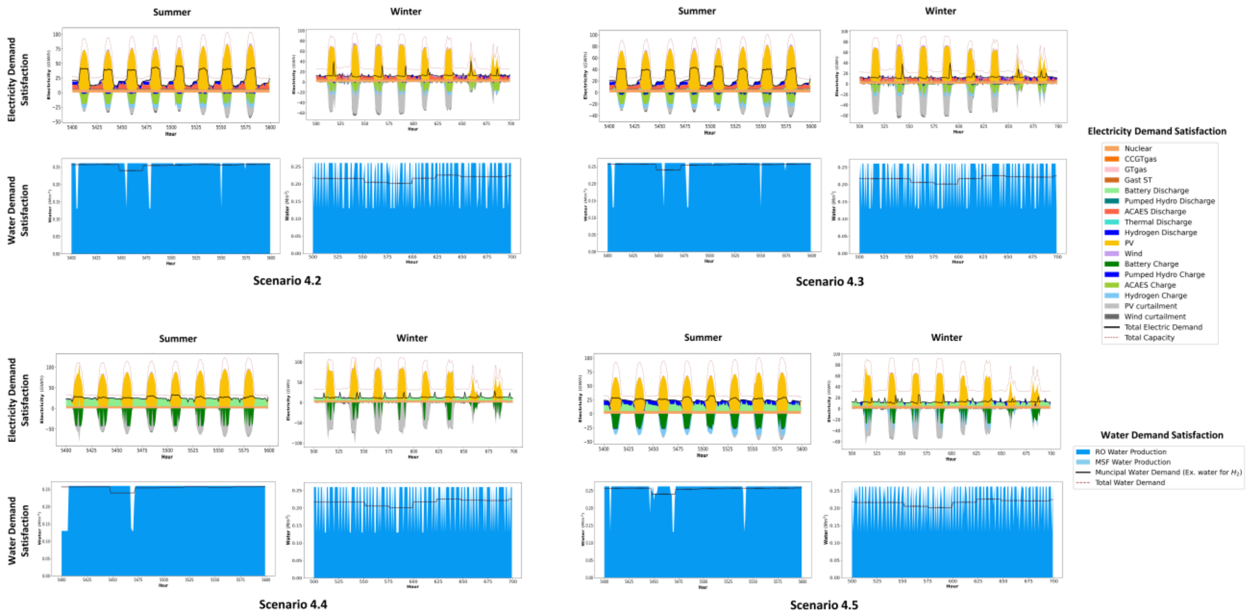

Figure S1.3.4 *Electricity and Water demand satisfaction figures in summer and winter across scenarios 4.2, 4.3, 4.4 and 4.5 outlined in Table S1.3, Related to Results and Discussion.*

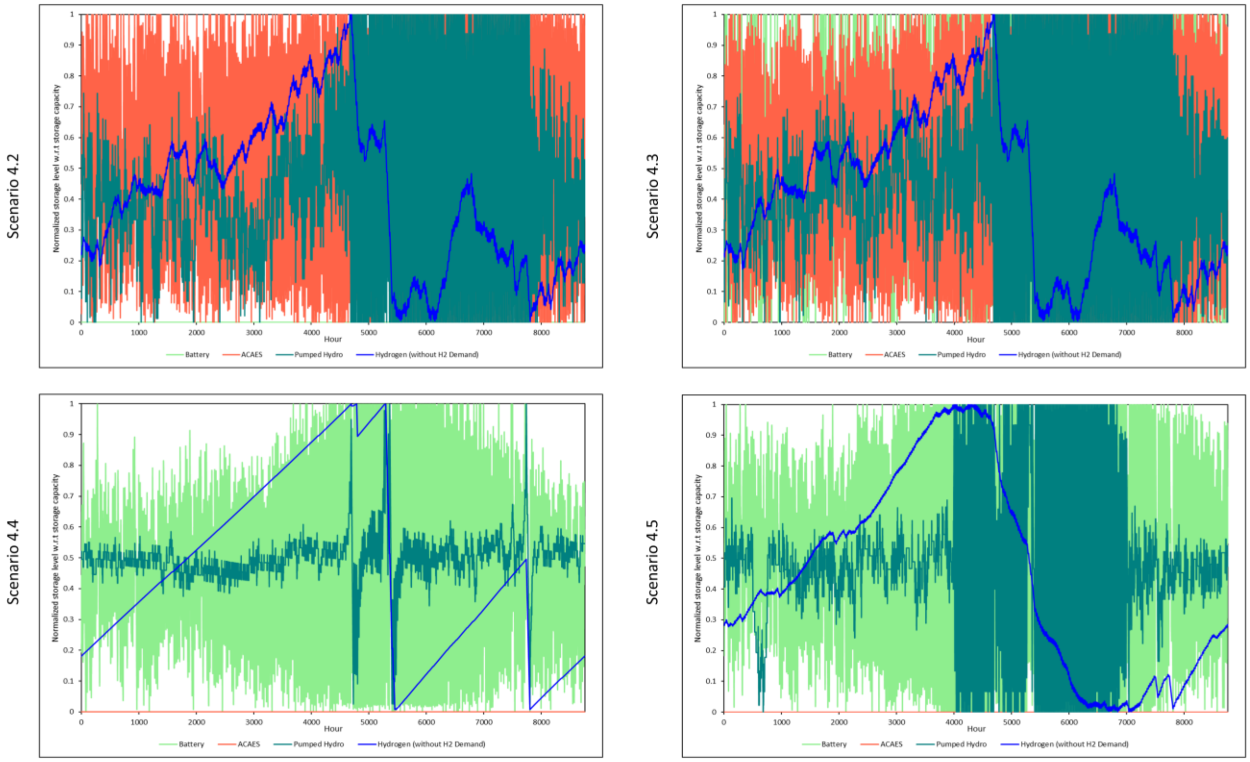

Figure S1.3.5 *Interplay of various storages as daily, weekly, and seasonal cycles for scenarios 4.2, 4.3, 4.4 and 4.5 outlined in Table S1.3, Related to Results and Discussion.*

Abbreviations

ACAES	Adiabatic Compressed Air Energy Storage
CCGT	Combined Cycle Gas Turbine
CSP	Concentrated Solar Power
GT	Open Cycle Gas Turbine
H_2	Hydrogen
MSF	Multistage Flash Thermal Desalination
PHS	Pumped Hydro Storage
PV	Solar Photovoltaic
ST	Steam Gas Turbine
TES	Thermal Energy Storage
TMY	Typical Meteorological Year

Subscripts

th	Thermal
cooling	Cooling
